# Basal ganglia neuropeptides show abnormal processing associated with L-DOPA-induced dyskinesia

**DOI:** 10.1038/s41531-022-00299-7

**Published:** 2022-04-13

**Authors:** Heather Hulme, Elva Fridjonsdottir, Theodosia Vallianatou, Reza Shariatgorji, Anna Nilsson, Qin Li, Erwan Bezard, Per E. Andrén

**Affiliations:** 1 Department of Pharmaceutical Biosciences, Medical Mass Spectrometry Imaging, Uppsala University, Uppsala, Sweden; 2 Science for Life Laboratory, Spatial Mass Spectrometry, Uppsala University, Uppsala, Sweden; 3 Motac Neuroscience, Manchester, M15 6WE UK; 4 Université de Bordeaux, Institut des Maladies Neurodégénératives, Bordeaux, France; 5 Centre National de la Recherche Scientifique, Institut des Maladies Neurodégénératives, Bordeaux, France

**Keywords:** Parkinson's disease, Parkinson's disease, Molecular imaging

## Abstract

L-DOPA administration is the primary treatment for Parkinson’s disease (PD) but long-term administration is usually accompanied by hyperkinetic side-effects called L-DOPA-induced dyskinesia (LID). Signaling neuropeptides of the basal ganglia are affected in LID and changes in the expression of neuropeptide precursors have been described, but the final products formed from these precursors have not been well defined and regionally mapped. We therefore used mass spectrometry imaging to visualize and quantify neuropeptides in 1-methyl-4-phenyl-1,2,3,6-tetrahydropyridine exposed parkinsonian and LID *Macaca mulatta* brain samples. We found that dyskinesia severity correlated with the levels of some abnormally processed peptides — notably, des-tyrosine dynorphins, substance P (1-7), and substance P (1-9) — in multiple brain regions. Levels of the active neuropeptides; dynorphin B, dynorphin A (1-8), α-neoendorphin, substance P (1-11), and neurokinin A, in the globus pallidus and substantia nigra correlated with putaminal levels of L-DOPA. Our results demonstrate that the abundance of selected active neuropeptides is associated with L-DOPA concentrations in the putamen, emphasizing their sensitivity to L-DOPA. Additionally, levels of truncated neuropeptides (which generally exhibit reduced or altered receptor affinity) correlate with dyskinesia severity, particularly for peptides associated with the direct pathway (i.e., dynorphins and tachykinins). The increases in tone of the tachykinin, enkephalin, and dynorphin neuropeptides in LID result in abnormal processing of neuropeptides with different biological activity and may constitute a functional compensatory mechanism for balancing the increased L-DOPA levels across the whole basal ganglia.

## Introduction

Parkinson’s disease (PD) is a neurodegenerative disorder characterized by motor symptoms including rigidity, bradykinesia, resting tremor, and shuffling gait. The primary pathological hallmark of PD is a loss of dopaminergic neurons in the substantia nigra pars compacta (SNc), leading to reduced dopamine levels in the striatum^1^. The most efficacious treatment for PD is administration of l-3,4-dihydroxyphenylalanine, (L-DOPA), the precursor of dopamine. However, prolonged use of L-DOPA leads to L-DOPA-induced dyskinesia (LID) in ~40% of patients after 4 years of treatment^2^. Although the mechanisms leading to LID are poorly understood, multiple neurochemical systems, including basal ganglia neuropeptides, have been associated with the condition^3,4^.

Dynorphin, enkephalin, and tachykinin neuropeptides of the basal ganglia have many effects, including regulation of neurotransmitter release, modulation of blood-brain barrier permeability, and stimulation of neuro-inflammatory responses^5–9^. The basal ganglia neuropeptides originate from the GABA (γ-aminobutyric acid)-ergic striatal spiny projection neurons (SPNs). Some striatal SPNs, known as indirect pathway SPNs, co-release enkephalin peptides in the external globus pallidus (GPe), while others known as direct striatal pathway SPNs co-release tachykinins (such as substance P and neurokinin A) and dynorphin peptides in the internal globus pallidus (GPi) and substantia nigra pars reticulata (SNr)^10,11^. In PD, the loss of dopamine in the striatum causes dysregulation of the basal ganglia circuit and thus affects downstream neuropeptide signaling.

In situ hybridization studies have consistently shown that PD and LID are associated with altered expression of neuropeptide precursor messenger ribonucleic acids (mRNA). Neuropeptide precursor mRNA is translated to prepropeptides such as proenkephalin (PENK), prodynorphin (PDYN), and protachykinin-1 (TKN1). These prepropeptides are then post-translationally processed by sequence-specific and tissue-specific enzymes to generate multiple processed neuropeptides that can have different biological activities^12^. Dopamine depletion is associated with increased expression of PENK mRNA while LID is associated with increased PDYN mRNA expression in striatal SPNs, in both rodent and primate models of PD^13,14^. Previously reported measurements of peptide levels are consistent with these changes in neuropeptide precursor expression patterns and suggest that some processed peptide sequences are affected more than others in a region-specific manner^4,15–17^. Using liquid chromatography mass spectrometry (LC-MS) analysis we showed that levels of dynorphin A and substance P neuropeptides were higher in the globus pallidus of dyskinetic primates than in non-dyskinetic controls^4^. Additionally, in rats, levels of the truncated peptide α-neoendorphin were higher in the 6-hydroxydopamine (6-OHDA) lesioned striatum following L-DOPA treatment and correlated with dyskinesia severity^15,16^.

Processed neuropeptides derived from the same precursor may have different receptor affinities and can mediate distinct effects^18^. The opioid peptides derived from PENK and PDYN bind to μ-, δ-, and κ-opioid receptors with different affinities. For example, met-enkephalins have high affinity for the δ-opioid receptor, while dynorphin A and α-neoendorphin have high affinity for κ-opioid receptors^19^. Substance P binds to the neurokinin-1 receptor, whereas neurokinin A has a high affinity for the neurokinin-2 receptor^12^. Furthermore, the μ- and κ-opioid receptors in both segments of the globus pallidus (GP) are negatively correlated with LID severity in a non-human primate model of PD^13^. Given the increased opioid tone associated with dyskinesia, the μ-opioid receptor, alone or in combination with other opioid receptors, has proved to be a promising target for alleviating dyskinesia in parkinsonian primates^20–23^. However, clinical efficacy has not been established yet. We recently showed that region-specific μ-opioid receptor agonism in the GPi ameliorated LID in a primate model of LID and another study identified a selective μ-opioid receptor antagonist that also reduced LID in the same model^20,21^. These studies illustrate the importance of fully understanding the neuropeptide alterations in LID, including their processed states and regional localization in the brain. Determining the effects of striatal L-DOPA and dopamine levels on neuropeptide levels in the output structures of the striatal SPNs may also shed light on the relationship between these two factors.

We have previously used matrix-assisted laser desorption/ionization mass spectrometry imaging (MALDI-MSI) to map regionally specific neuropeptide changes in the unilateral 6-OHDA lesioned rat model of PD and those that occur during L-DOPA treatment^16^. In the study presented here, we used this technique to characterize neuropeptide alterations in a pre-existing cohort of samples from the 1-methyl-4-phenyl-1,2,3,6-tetrahydropyridine (MPTP) non-human primate model of PD during the peak LID behavior period^24–27^. This model targets depletion of the catecholaminergic neurons and closely mimics the clinical symptoms of PD and LID^28,29^. We have previously used the same animals to image catecholamine and indolamine neurotransmitters and their metabolites using MALDI-MSI^27^ and to analyze expression profiles of neuropeptides using LC-MS^4^. Here we take advantage of MALDI-MSI, which makes it possible to accurately and simultaneously determine the tissue distributions of multiple neuropeptides, enabling their quantitative regional and sub-regional mapping.

## Results

### Neuropeptide identification

The neuropeptides were identified using accurate masses obtained through ultrahigh resolution Fourier-transform ion cyclotron resonance (FTICR) MS and LC-MS/MS as previously described^16^. Twenty-three neuropeptides were imaged using MALDI-MSI: four from TKN1, 11 from PENK, and seven from PDYN (Supplementary Table 1). Although not a classical neuropeptide, PEP-19 is a small calmodulin-binding protein that was reported to be significantly decreased in a PD mouse model and has been suggested to play a role in neurotransmitter release^30,31^. Therefore, levels of PEP-19 were also analyzed.

### Multivariate analysis of neuropeptides

**Fig. 1:**
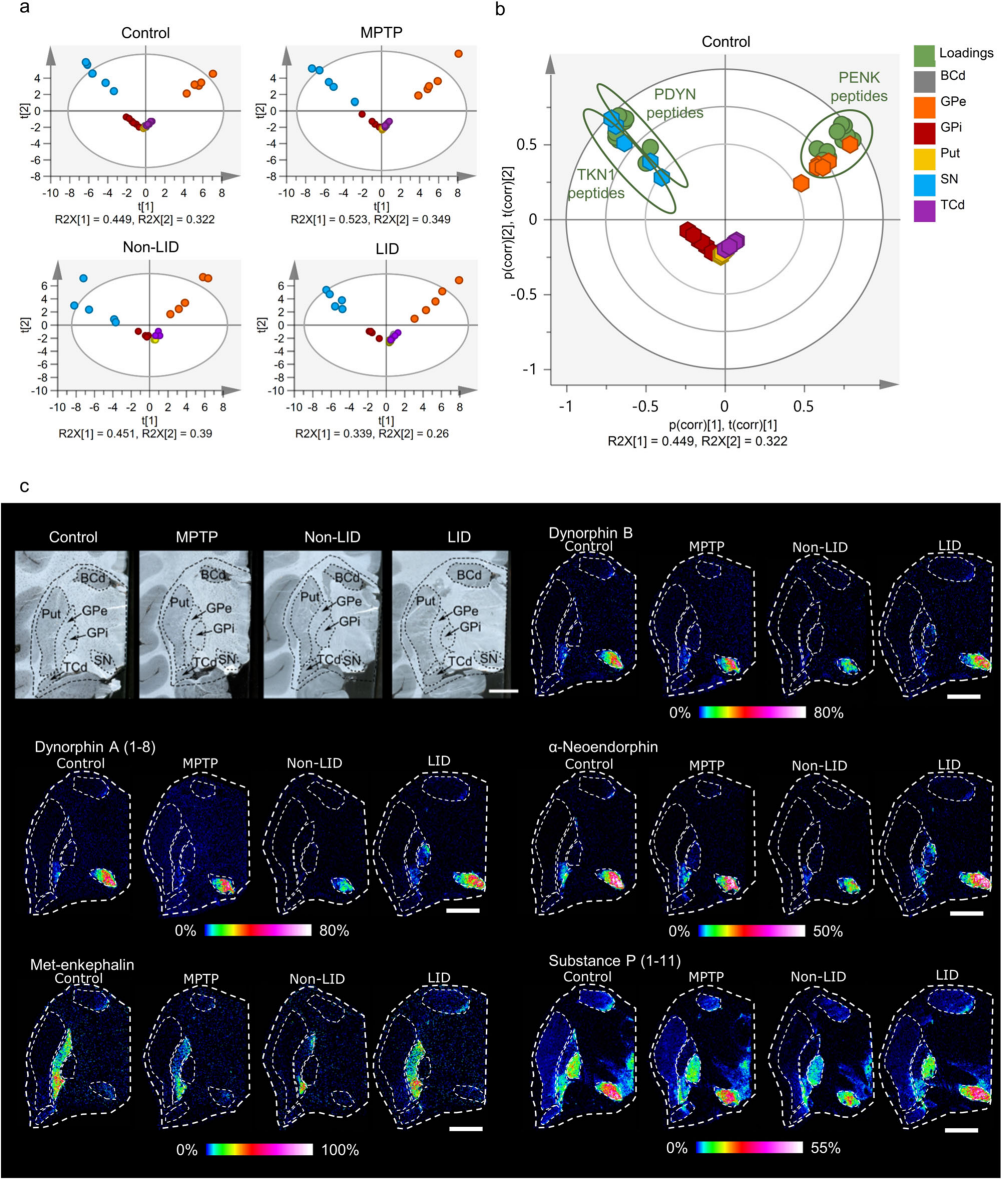
Regional distribution of neuropeptides in the basal ganglia. **a** Principal component analysis (PCA) score plots showing regional abundances of detected neuropeptides in primate brains in the four experimental groups: MPTP-treated (MPTP), MPTP + L-DOPA-treated without LID (non-LID), MPTP + L-DOPA-treated with LID (LID), and saline-treated (controls). Score plots with scores color-coded according to regions. **b** Biplot showing scores and loadings of the PCA for the control group, with loadings (green) annotated according to the peptide precursor. The BCd scores are located behind the TCd and Put scores and are therefore not visible on the plots. **c** Optical images of brain sections from the four groups and representative MALDI-MSI ion images (from the same sections shown in the optical images) of signaling neuropeptides derived from the three precursors: DYN, Dyn B, Dyn A (1-8), and α-neoendorphin; PENK, met-enkephalin; and TKN1, SP (1-11). Scale bars, 5 mm; lateral resolution, 100 μm. All MALDI MS images are RMS-normalized, and intensities are log-transformed and indicated with a rainbow color scale, scaled to specific percentages as annotated below each image. The optical images show basal ganglia at the -6 mm ac level^51^. ac anterior commissure, BCd body of the caudate nucleus, GPe external globus pallidus, GPi internal globus pallidus, Put putamen, SN substantia nigra, TCd tail of the caudate nucleus.

Brains sections from animals in four treatment groups were imaged by MALDI-MSI. The groups were: (i) controls, receiving only saline administration (*n* = 6); (ii) MPTP, receiving daily MPTP injections until stable parkinsonism became apparent (*n* = 5); (iii) non-LID, receiving MPTP until stable parkinsonism followed by 3 months of L-DOPA treatment without showing dyskinesia (*n* = 6); and (iv) LID, receiving MPTP until stable parkinsonism followed by 3 months of L-DOPA treatment resulting in dyskinesia (*n* = 6). Principal component analysis (PCA) was then used to explore the variation in neuropeptide levels in brain regions of animals in each group. Statistical test results for the analyses are shown in Supplementary Tables 2–4. The PCA scores for specific brain regions of animals within each group were very similar (Fig. 1a), indicating high similarity in their neuropeptide profiles. The substantia nigra (SN) and GPe were widely separated from each other in the score plots, and distinctly separated from the other regions along the first principal component (t[1]). These results show that the neuropeptide profiles of the SN and GPe are different, and they are also both different from the other regions analyzed. The striatal regions, the caudate body (BCd), the caudate tail (TCd), and the putamen (Put) clustered together, indicating high similarity in their peptide profiles. The GPi was located in close proximity to the striatal regions but was closer to the SN, while the striatal regions were closer to the GPe. Factors responsible for the regions’ separation were visualized using score and loading biplots, which showed that the main output regions of the striatal SPNs involved in the direct pathway (the SN and GPi) had high levels of peptides derived from PDYN and TKN1, although the SN clearly had higher levels of these peptides than the GPi (Fig. 1b). The GPe had high levels of PENK peptides, in accordance with their involvement in the indirect pathway (Fig. 1b).

MALDI-MS images of representative peptides derived from PDYN, PENK, and TKN1 showed that they had distinct regional distributions (Fig. 1c). The PDYN-derived signaling peptides α-neoendorphin (α-neo), dynorphin (Dyn) A (1-8), and Dyn B were most abundant in the SN and their levels were elevated in the GPi of the LID animals (Fig. 1c). TKN1-derived substance P (SP) (1-11) was most abundant in the SN and GPi, while the PENK-derived met-enkephalin was highly abundant in the GPe (Fig. 1c).

**Fig. 2:**
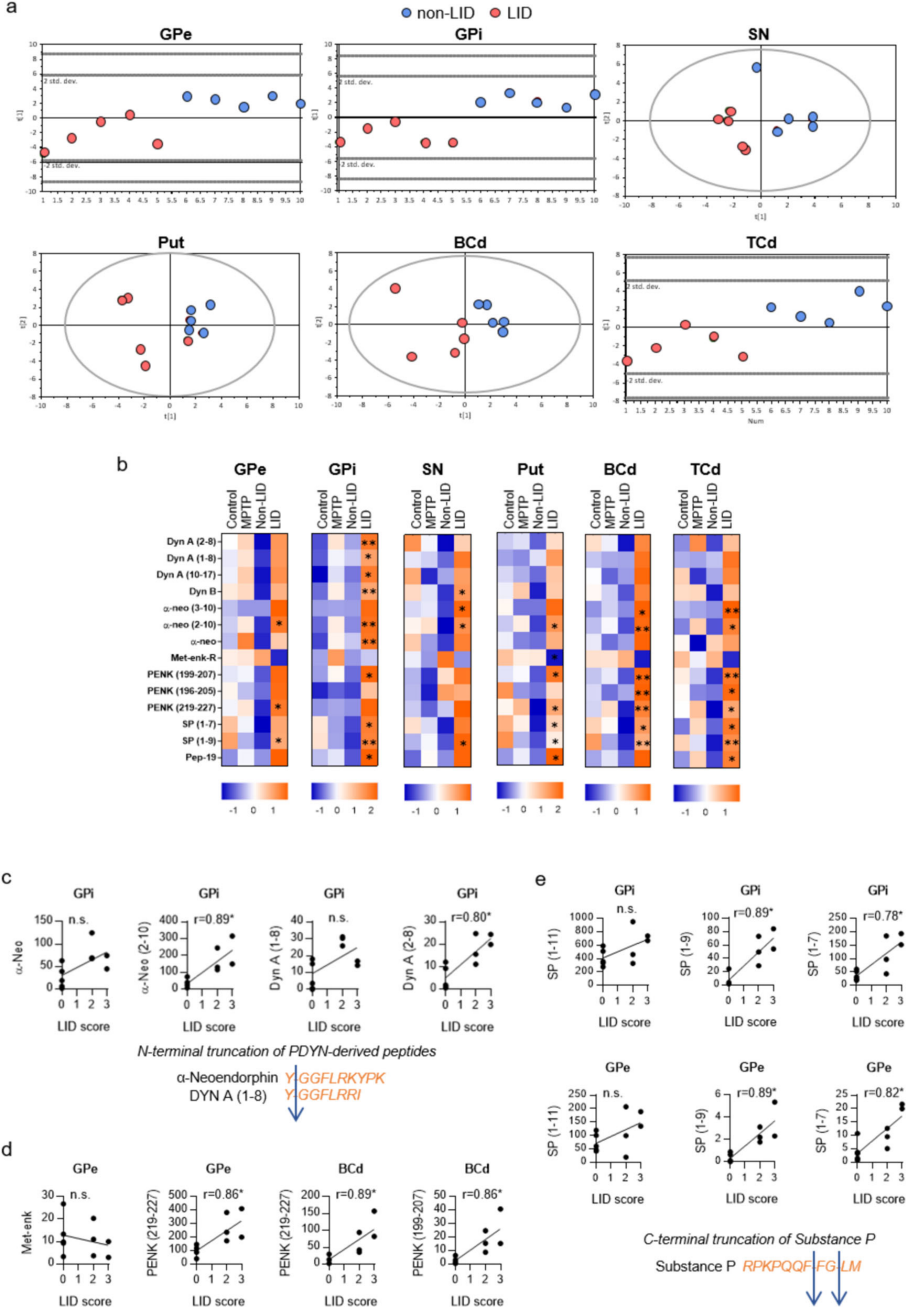
Neuropeptide alterations associated with LID. **a** Partial least squares-discriminant analysis (PLS-DA) score plots of neuropeptides in six basal ganglia regions of non-LID (blue) and LID (red) primates. **b** Heat maps showing z-scores of peptides with variable importance in projection values (VIP) > 1 for each experimental group in the six basal ganglia brain regions. The Mann–Whitney test with false discovery rate (FDR) correction was used to test the significance of differences in neuropeptide levels between non-LID and LID samples, *N* = 5, *P ≤ 0.05; **P ≤ 0.01. **c**–**e** Analyses of the correlations between LID scores and ion intensities of **c** PDYN-derived peptides, **d** PENK-derived peptides, and **e** TKN1-derived peptides in specific brain regions. Samples from non-LID and LID animals were included in the analysis, *N* = 10; r represents the Spearman’s correlation coefficient; *P ≤ 0.05; **P ≤ 0.01.

PCA was also used to obtain an overview of neuropeptides in six basal ganglia regions (Put, SN, TCd, BCd, GPi, and GPe) in the four groups (Supplementary Fig. 1). The plots show each biological replicate as a point on the PCA plot. Because all 23 analyzed neuropeptides were included as dependent variables in the PCA analysis, the plots provide an overview of the differences between the neuropeptide profiles of each animal. The color of each point indicates the treatment applied to the corresponding animal. There was no separation of control and MPTP groups in the resulting score plot, but there was some separation of the non-LID and LID groups in the GPi, GPe, and Put regions (Supplementary Fig. 1). The scores of one LID animal did not cluster with the corresponding scores of other LID animals for several brain regions. Therefore, data pertaining to this animal were excluded from the partial least square discriminant analysis (PLS-DA), whose primary purpose was to elucidate differences between the LID and non-LID animals, and from the analyses presented in Fig. 2 and Fig. 4. To identify peptides associated with dyskinesia, the profiles of six brain regions (GPe, GPi, SN, Put, BCd, and TCd) of the non-LID and remaining LID animals were then subjected to PLS-DA (Fig. 2a). Fifteen peptides had variable importance in projection (VIP) coefficients greater than 1, indicating a significant contribution to the separation of the groups, and were therefore further analyzed using Mann–Whitney tests with false discovery rate (FDR) correction (Supplementary Table 2). The neuropeptides exhibiting significant differences between non-LID and LID groups in various brain regions were Dyn A (2-8), Dyn A (1-8), Dyn A (10-17), Dyn B, α-neo, α-neo (3-10), α-neo (2-10), met-enkephalin-R (met-enk-R), PENK (199-207), PENK (196-205), PENK (219-227), SP (1-7), SP (1-9), and the calmodulin-binding peptide PEP-19 (48-62) (Fig. 2b and Supplementary Table 2).

### Changes in PDYN-derived peptides

Levels of dynorphin neuropeptides differed most strongly between the LID and non-LID animals, and the differences were strongest in the GPi, where levels of six dynorphin neuropeptides were higher in the LID group (Fig. 2b). These neuropeptides were Dyn A (2-8), Dyn A (1-8), Dyn A (10-17), α-neo, α-neo (2-10), and Dyn B. The α-neo (2-10) peptide exhibited the most pronounced difference, and was more abundant in the LID group than in the non-LID group in all studied brain regions. The α-neo (3-10) and Dyn B peptides were also more abundant in multiple brain regions of the LID animals.

To further elucidate relationships between the neuropeptides and severity of dyskinesia, we examined the correlations between their abundances in specific brain regions and dyskinesia scores. Significant correlations were observed for two N-terminal truncated α-neo derivatives: α-neo (2-10) in the GPe, GPi, BCd and SN, and α-neo (3-10) in the GPi and BCd. Conversely, dyskinesia scores did not correlate significantly with levels of the full α-neo signaling peptide (Fig. 2c and Supplementary Table 3). Similarly, levels of the signaling peptide Dyn A (1-8) did not correlate with dyskinesia scores, but levels of its N-terminal truncated derivative Dyn A (2-8) in the GPi correlated significantly with dyskinesia severity (Fig. 2c and Supplementary Table 3).

### Changes in PENK-derived peptides

The PENK-derived peptides that were affected by dyskinesia in the greatest number of brain regions were PENK (219-227) and PENK (199-207) (Fig. 2b). Compared to the corresponding regions in non-LID animals, PENK (219-227) levels were higher in the Put, GPe, BCd, and TCd of LID animals, while PENK (199-207) levels were higher in the GPi, Put, BCd, and TCd (Fig. 2b). Dyskinesia severity correlated significantly and positively with PENK (219-227) levels in the GPe and BCd, and PENK (199-207) levels in the BCd (Fig. 2d and Supplementary Table 3).

### Changes in TKN1-derived peptides

Levels of tachykinin neuropeptides were higher in the LID group than in non-LID animals in multiple regions (Fig. 2b). Levels of the full-length neuropeptide SP (1-11) did not differ significantly between the experimental groups in any of the analyzed regions. However, its C-terminal truncated derivatives SP (1-9) and SP (1-7) were more abundant in many brain regions of the dyskinetic animals (Fig. 2b). Specifically, SP (1-9) was more abundant in all investigated brain regions of LID animals while SP (1-7) was more abundant in the Put, GPi, BCd, and TCd. Interestingly, correlation analysis revealed that dyskinesia scores correlated significantly and positively with levels of both truncated SP products in both segments of the GP, and with levels of SP (1-9) in the BCd. However, no significant correlations were observed for SP (1-11) (Fig. 2e and Supplementary Table 3).

### MALDIMSI of abnormally processed neuropeptides in non-LID and LID animals

**Fig. 3:**
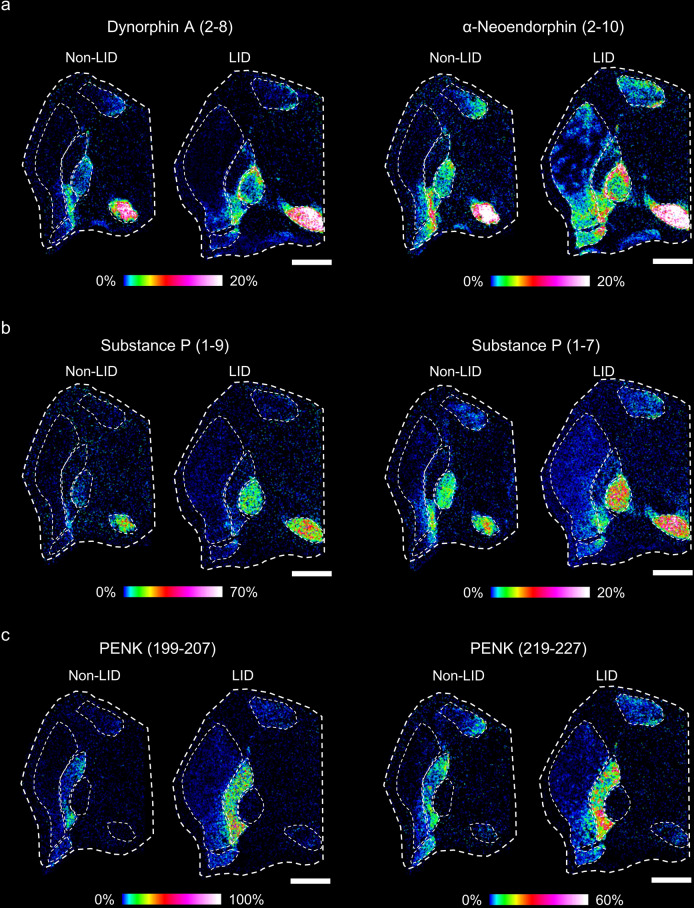
Processed fragments of active neuropeptides elevated in LID. MALDI MS images of **a** des-tyrosine dynorphin neuropeptides, Dyn A (2-8) and α-neoendorphin (2-10), **b** processed substance P peptides (1-9) and (1-7), and **c** PENK-derived peptide sequences, PENK (199-207) and PENK (219-227) without known biological functions. All images are RMS-normalized, and intensities are log-transformed and indicated with a rainbow color scale, scaled to specific percentages as annotated below each MALDI MS image. Scale bars, 5 mm; lateral resolution, 100 μm.

The MALDI-MS images revealed that levels of the N-terminal truncated peptides Dyn A (2-8) and α-neo (2-10) were higher in LID animals than in non-LID animals (Fig. 3a). The distribution of α-neo (2-10) in the Put resembled that in the striosomes^32^. Levels of the C-terminal truncated SP derivatives SP (1-7) and SP (1-9) and PENK-derived peptides lacking the enkephalin motif (YGGF), i.e., PENK (199-207) and PENK (219-227), were higher in multiple regions of LID animals than in the corresponding regions of non-LID animals (Fig. 3b, c).

### Single doses of L-DOPA in MPTP-exposed animals

We next investigated the impact of a single dose of L-DOPA on neuropeptide levels in the GPi in MPTP-exposed animals. The GPi region showed the greatest number of neuropeptide changes when comparing non-LID to LID. In addition, it had the highest number of neuropeptides correlated to LID scores and the PCA results showed that the neuropeptide profile of the GPi closely resembles those of the striatal regions and SN. We therefore investigated this brain region further. The neuropeptide profile of the singly L-DOPA dosed MPTP-administered animals (*n* = 6) resembled that of the non-LID animals (Supplementary Fig. 2), suggesting that initial L-DOPA treatment did not induce changes in neuropeptide levels in the GPi. It thus appears that increases in the levels of these peptides are specifically related to dyskinesia and occur only after prolonged L-DOPA treatment.

### Sub-regional analysis of neuropeptides

**Fig. 4:**
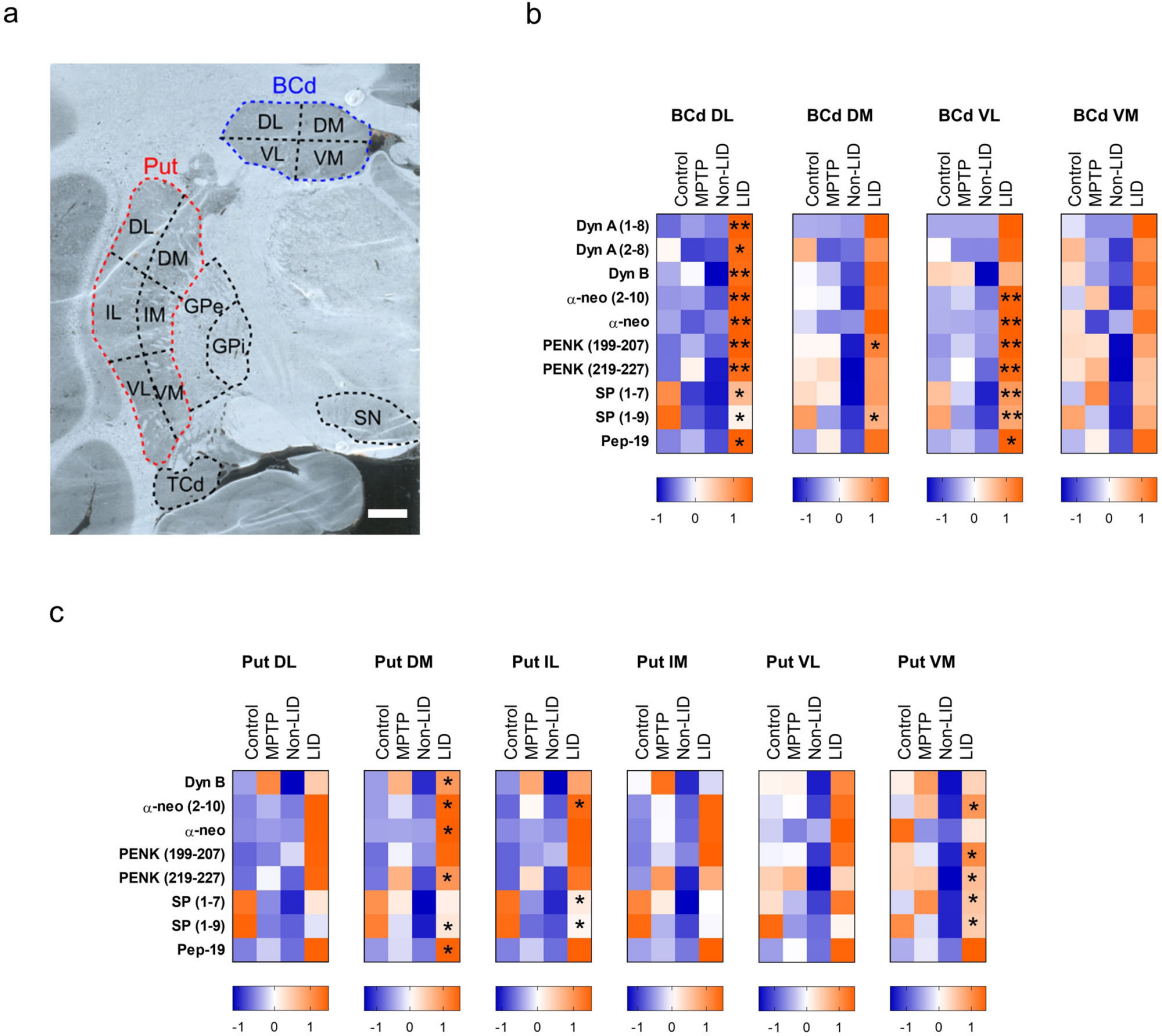
Neuropeptide alterations in sub-regions of caudate and putamen associated with LID. **a** Optical image of a macaque brain section at ac -6 mm^51^, showing subregional divisions of the Put and BCd. **b**, **c** Heat maps showing z-scores for each group of peptides with variable importance in projection values (VIP) > 1 in sub-regions of the BCd and Put, respectively. Asterisks indicate the significance of differences in neuropeptide levels between non-LID and LID animals according to the Mann–Whitney test with the false discovery rate (FDR) correction (*N* = 5, *P ≤ 0.05; **P ≤ 0.01). The scale bar is 2 mm. BCd body of the caudate nucleus, DL Dorsolateral, DM dorsomedial, GPe external globus pallidus, GPi internal globus pallidus, IL intermediolateral, IM intermediomedial, Put putamen, SN substantia nigra, TCd tail of the caudate nucleus, VL ventrolateral, VM ventromedial.

The neuropeptides’ distributions varied across the analyzed regions. Previous studies on the functional anatomy of the striatum have defined sub-regions of the Put and BCd according to the areas of the cortex they are connected to and their roles. Therefore, we tested the hypothesis that the observed treatment-related elevations in neuropeptide levels may have been particularly strong in specific sub-regions of the striatal brain areas, Put, and BCd (Fig. 4a). Briefly, in the BCd, significant between-group differences were primarily found in the dorsolateral (DL) and ventrolateral (VL) sub-regions (Fig. 4b). We also detected subregion-specific differences in the levels of several neuropeptides in the putamen, mostly in the dorsomedial (DM) and ventromedial (VM) sub-regions (Fig. 4c). Further information can be found in Supplementary Note.

### Correlation of neuropeptides with L-DOPA and dopamine

**Fig. 5:**
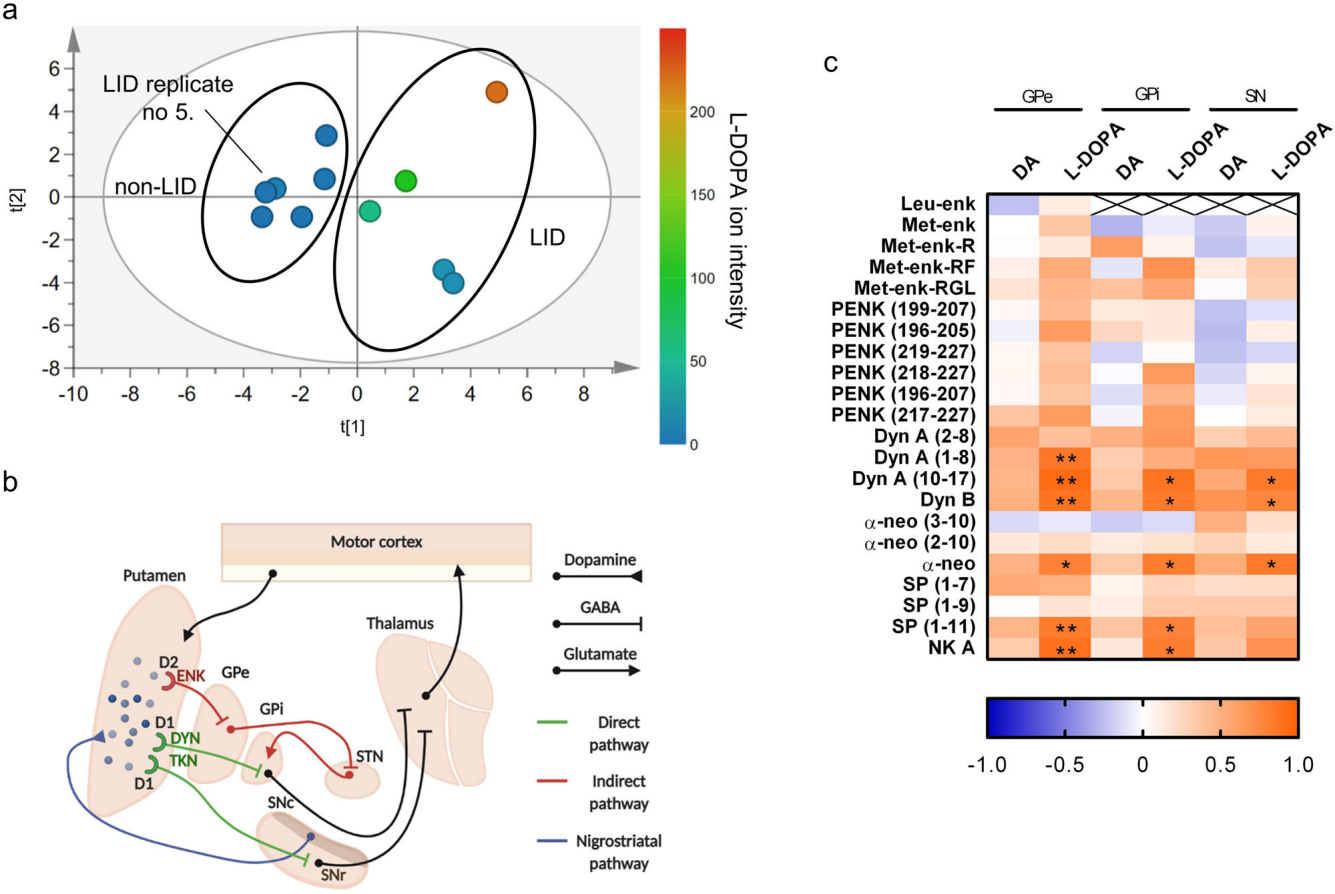
Relationship between the abundance of L-DOPA and the basal ganglia neuropeptides. **a** Principal component analysis (PCA) score plot based on neuropeptide abundance in the GPi of samples from non-LID and LID groups. Scores are color scaled according to L-DOPA ion intensity in samples from each animal. The two ovals enclose samples belonging to non-LID and LID groups with the exception of biological replicate 5 from the LID group, which clustered with non-LID samples. **b** Connectivity of the basal ganglia showing the projections of the neuropeptide-co-releasing spiny projection neurons (SPNs) from the putamen to the GPe, GPi, and SN. **c** Results of Pearson’s correlation analysis of putaminal dopamine and L-DOPA levels with neuropeptides in output regions of the SPNs. Heat maps are color-coded according to Pearson’s correlation coefficients, *N* = 11. Red and blue colors indicate positive and negative correlations, respectively (Supplementary Table 4). Asterisks indicate significant correlations: *P ≤ 0.05; **P ≤ 0.01. ENK enkephalin peptides, DYN dynorphin peptides, GABA γ-aminobutyric acid, GPe external globus pallidus, GPi internal globus pallidus, DA dopamine, SN substantia nigra, SNc substantia nigra pars compacta, SNr substantia nigra pars reticulata, STN subthalamic nucleus, TKN1 tachykinin peptides.

We next investigated potential correlations between neuropeptide levels and those of L-DOPA and dopamine. We included data for LID biological replicate five, which was excluded from the preceding analyses, because we wanted to determine whether the low neuropeptide levels observed in this replicate might be related to differences in its levels of L-DOPA and dopamine. The PCA of neuropeptide levels in the GPi (see Fig. 5a) separated the data for the LID and non-LID animals with the exception of LID biological replicate five, which clustered with the non-LID animals. In Fig. 5a, the results for individual biological replicates are color-coded based on their L-DOPA levels, which were determined in a previously reported analysis of the relative abundances of L-DOPA, multiple neurotransmitters, and related metabolites in the same brain region and animals used in this work^27^. Samples with high levels of L-DOPA were located on the right-hand side of the PCA score plot while those with low levels (indicated by a dark blue coloration) were located on the left side, indicating a separation along the first principal component (t[1], Fig. 5a). Moreover, biological replicate 5 of the LID group had low levels of L-DOPA in the brain and clustered with the non-LID group. Replicates within the LID group were also separated along the second principal component (t[2]) based on their L-DOPA levels.

Since the brain levels of L-DOPA could, at least partially, explain the separation of the non-LID and LID groups with respect to neuropeptide levels in the GPi (Fig. 5a), we investigated the relationships between neuropeptide levels and those of L-DOPA and dopamine more closely. To this end, we analyzed correlations between the relative abundances of dopamine and L-DOPA in the Put of non-LID and LID animals and the neuropeptide levels in the three output structures of the striatal SPNs, i.e., the GPe, GPi, and SN (Fig. 5b). Interestingly, putaminal L-DOPA levels but not dopamine levels correlated positively with several PDYN and TKN1 peptides in the three output regions (Fig. 5c and Supplementary Table 4). Three PDYN-derived peptides— α-neo, Dyn A (10-17) and Dyn B—correlated with L-DOPA in all output regions (Fig. 5c and Supplementary Table 4). In addition, Dyn A (1-8) in the GPe correlated with putaminal L-DOPA levels. SP (1-11) and neurokinin A levels in the GPe and GPi correlated positively with putaminal L-DOPA levels (Fig. 5c and Supplementary Table 4).

## Discussion

L-DOPA treatment has been shown to strongly influence the expression of neuropeptide precursor proteins and abundance of basal ganglia neuropeptides^4,13,15,16^, but our knowledge of their processed states and involvement in LID remains limited. In this study we conducted a comprehensive analysis of 23 peptides in six basal ganglia regions and made three major observations concerning the effects of LID on these neuropeptides.

Firstly, LID was associated with a widespread increase in the levels of multiple neuropeptides across all six basal ganglia structures whereas non-LID animals had low levels of these neuropeptides even when compared to the control and MPTP groups. Secondly, the severity of LID (based on LID scores) correlated with the abundance of processed derivatives of the active neuropeptides in the GP, SN, and BCd – specifically, derivatives formed by N-terminal cleavage of PDYN-derived neuropeptides and C-terminal cleavage of SP peptides. Finally, putaminal levels of L-DOPA (but not dopamine) correlated with the abundance of the active neuropeptides Dyn A (1-8), Dyn B, α-neo, SP (1-11), and neurokinin A in the output structures of the basal ganglia. However, unlike the LID scores, they did not correlate with the levels of processed derivatives. The last observation suggests that striatal L-DOPA itself, without being converted to dopamine, may induce neuropeptide signaling in the output structures of the SPNs^27,33,34^.

According to the classical view of basal ganglia function in PD, dopamine depletion results in hyper-activation of the indirect pathway striatal SPNs that co-release enkephalin peptides in the GPe^10,11^. In LID the balance is shifted towards hyper-activation of the direct pathway striatal SPNs, which co-release dynorphins and tachykinins in the GPi and SNr^10,11^. However, studies on non-human primates have found that the organization of the basal ganglia is less segregated than previously thought and the striatofugal neurons project to both parts of the GP, albeit to different extents^35,36^, which is consistent with our data. Specifically, we measured increases in levels of dynorphins and tachykinins in the GPe, the brain region associated with the indirect pathway, along with increased levels of PENK peptides in the GPi and SN, which are associated with the direct pathway. In addition, many previous publications have reported increased dynorphin signaling associated with LID, prompting a tendency to focus on the opioid system^13,14,37,38^. Although we found that the biggest effect in LID was related to dynorphin peptides, our data suggest abnormal signaling via peptides derived from all three precursors (PDYN, TKN1, and PENK), highlighting the involvement of the tachykinin system as well as the opioid system, which is in agreement with our earlier LC-MS study^4^. Accordingly, studies on the 6-OHDA rat model of LID have shown that antagonism of the neurokinin 1 (NK 1) receptor (the SP receptor) alleviates LID without affecting the anti-parkinsonian action of L-DOPA^39,40^.

The most pronounced neuropeptide alteration observed in this study was that levels of α-neo (2-10), the des-tyrosine form of α-neo^41^, were elevated in all studied brain regions in dyskinetic animals. Very few studies have focused on this neuropeptide and its function remains unknown. Our results are generally consistent with those of an earlier MALDI-MSI study showing that levels of several PDYN-derived peptides, including α-neo (2-10), were elevated in a 6-OHDA-lesioned rat model of LID and correlated with LID severity, whereas SP (1-11) did not correlate with LID^15^. However, the two shorter SP-derived peptide sequences SP (1-9) and SP (1-7) were not detected in the earlier study^15^. Conversely, in this work we detected correlations between LID scores and the levels of both SP (1-7) and SP (1-9) in the GPi and GPe. We also found that LID scores correlated significantly with levels of PENK (199-207) in the BCd and levels of PENK (219-227) in the GPe and BCd, but not with levels of Leu-enkephalin (YGGFL) or Met-enkephalin (YGGFM). Taken together, these results suggest that increases in tone of tachykinin, enkephalin, and dynorphin neuropeptides result in abnormal processing of neuropeptides across the whole basal ganglia in LID. Synaptic release of neuropeptides is followed by postsynaptic processing, which is mainly performed by neprilysin, angiotensin converting enzyme and aminopeptidase N, enzymes involved in processing a wide range of peptide hormones^18^. It is reasonable to hypothesize that these enzymes may be responsible for producing the processed peptides whose abundance was found to correlate with LID scores in this work.

The basal ganglia neuropeptides that are generally regarded as active, fully processed neuropeptides include opioid peptides containing the sequence YGGF such as the PENK-derived met-enkephalin, extensions of this peptide (YGGFM-R, YGGFM-RF, YGGFM-RGL), and leu-enkephalin, which can also be derived from PDYN along with Dyn A (1-17), Dyn B (1-13), and α-neo (1-10). In addition, the tachykinins SP (1-11) and neurokinin A (1-10) are considered fully processed peptides that act on neurokinin receptors. Cleavage of the N-terminal tyrosine from enkephalin peptides is thought to deactivate the peptides^42^. In addition, C-terminal cleavage of SP weakens its typical activity^43^ or changes its biological profile. Our previous studies using in vivo microdialysis and LC-MS showed that SP 1-7 and SP 1-9 are the two most abundant metabolic N-terminal fragments in the striatum^44^. SP (1-7) was reported to act on an unknown receptor that is distinct from the NK 1 receptor and any of the known opioid or tachykinin receptors^12^. Additionally, incubation with SP 1-9 increased the endogenous dopamine outflow in rat striatal brain slices. This increase was reversed by the muscarinic antagonist atropine but not by an NK 1 antagonist, and co-incubation with SP and SP 1-9 revealed a negative interaction between the parent peptide and its fragments^7^. The interactions of SP 1-7 and SP 1-9 with receptors may thus differ significantly from those of the parent peptide SP.

We found that levels of neuropeptide products formed by both types of cleavage correlated with dyskinesia, possibly reflecting counteractive responses to the high concentrations of basal ganglia neuropeptides in the dyskinetic state. However, the effects of the further processed peptides observed in this study on basal ganglia activity remain largely unknown. The des-tyrosine form of Dyn A has been shown to act via non-opioid mechanisms^45,46^ that include reversing the effects of the nicotinic acetylcholine receptor inhibitor mecamylamine, suggesting a potential role in the acetylcholine system^47^. It is thus reasonable to hypothesize that other des-tyrosine peptides such as α-neo (2-10) may also have non-opioid activity and could play some role in LID.

Our final observation that L-DOPA levels in the putamen correlate with several neuropeptide levels in the SPNs output structures suggests that L-DOPA may affect neuropeptide signaling without being converted to dopamine. Furthermore, an LID replicate with unusually low L-DOPA levels had neuropeptide levels similar to non-LID animals. L-DOPA is typically administered orally to patients and thus competes with dietary neutral amino acids for transport across both the gut and blood-brain barriers. In addition, it is subject to drug metabolism before reaching the brain; consequently, oral administration can lead to highly variable dosing of L-DOPA in the putamen. It is possible that the animal with low levels of L-DOPA in the putamen had different BBB or gut permeability properties that affected the levels of L-DOPA reaching the putamen. Our results indicate that such inconsistent levels of L-DOPA in the basal ganglia can have a direct effect on neuropeptide signaling systems, highlighting the need to better understand how fluctuating L-DOPA levels in patients affect the neuropeptide system and related PD and LID symptoms.

In summary, LID is associated with elevated levels of peptides derived from all three neuropeptide precursors involved in the basal ganglia circuit: PDYN, TKN1, and PENK. Levels of truncated peptides, particularly those associated with the direct pathway (i.e., dynorphins and tachykinins), correlate with dyskinesia severity. In addition, levels of the neuropeptides Dyn A (1-8), Dyn B, α-neo, and NK A in output structures of the direct and indirect pathway SPNs correlate with levels of L-DOPA in the putamen, underlining their sensitivity to L-DOPA. Pharmacological modulation of neuropeptide signaling has been a desirable non-dopaminergic treatment target for LID in the past 10-15 years, but its clinical effectiveness remains limited^20^. Further investigations into the abnormal neuropeptide processing that alters the biological profiles of the processed peptides and the effect of L-DOPA on neuropeptide expression and biotransformation are needed to fully understand the role these systems play in PD and LID and their potential as targets for pharmacological intervention.

## Methods

### Chemicals

Acetonitrile, water, trifluoroacetic acid (TFA), and chloroform were purchased from VWR (Stockholm, Sweden). 2,5-Dihydroxybenzoic acid (DHB) was purchased from Merck (Darmstadt, Germany). FMP-10 reactive matrix was synthesized in-house as previously described^48^.

### Ethical statement

Experiments were carried out in accordance with the European Communities Council Directive of November 24, 1986 (86/609/EEC) regarding care of laboratory animals in an AAALAC-accredited facility following acceptance of the study design by the Institute of Lab Animal Science (Chinese Academy of Science, Beijing, China) IACUC.

### Animal experiments

Experiments were performed on tissue from a previously published brain bank^24–27^. Female rhesus monkeys (*Macaca mulatta*: mean age = 5 ± 1 years, mean weight = 5.3 ± 0.8 kg, *n* = 29) were randomly assigned to treatment groups. Six were kept as controls and treated with saline injections, while the remainder received a daily dose of MPTP (0.2 mg/kg, i.v., Sigma, St Louis, MO), to induce parkinsonian symptoms, according to a previously published protocol^28,29^. Following stabilization of the MPTP-induced symptoms, animals received either saline (MPTP, n = 5) or L-DOPA, n = 12, (non-LID and LID) twice daily for three months (20 mg/kg, p.o.). A subgroup of MPTP exposed animals (n = 6) received a single dose of L-DOPA one hour prior to euthanasia (MPTP + single L-DOPA). The animals were rated daily for parkinsonian symptoms and dyskinesia using a PD clinical rating scale optimized for macaques^24,26,49^, and a dyskinesia disability scale^50^. The mean PD scores of the non-LID and LID groups before L-DOPA treatment were 10.2 and 9.3, respectively, with the minimal disability score being zero and the maximum being 25. Based on the dyskinesia scores, the animals were divided into a non-LID group (*n* = 6) comprising animals scoring zero and a LID group (*n* = 6) comprising animals scoring above zero (the mean score for these animals was 2.3 out of 4, indicating moderate dyskinesia). Animals were euthanized with an overdose of pentobarbital (150 mg/kg) one hour after the last L-DOPA dose. Brains were quickly removed after death, immediately frozen in dry ice-cooled isopentane, and then stored at −80 °C. The time between euthanasia and freezing of the brains was 10 min in all cases.

### Tissue sample preparation for MALDI-MSI

The brain samples were sectioned using a cryostat microtome (Leica CM3050S cryostat, Leica Microsystems, Wetzlar, Germany) to a thickness of 12 μm. The sections were thaw-mounted onto conductive indium tin oxide-coated glass slides (Bruker Daltonics, Bremen, Germany) and stored at −80 °C until analysis.

Samples for MALDI-MSI of neuropeptides were prepared according to a previously published protocol^16^. Briefly, slides were removed from −80 °C storage and immediately thawed by warming their undersides while drying under a stream of nitrogen. The slides were further dried in a desiccator for 30 min. To remove lipids from the tissue, which suppress ionization of neuropeptides, each slide was washed in 45 ml of chloroform for 30 s. The slides were then immediately dried in a desiccator for 15 min. For matrix preparation and application, 25 mg/ml of DHB was dissolved in a solution of 50% acetonitrile, 50% water and 0.2% TFA. The matrix was applied using an automated matrix sprayer (TM-sprayer, HTX technologies) in four passes at 85 °C (with gas pressure 6 psi, pump flow rate 120 μl/min, nozzle velocity 1200 mm/min, and 2 mm track spacing).

Samples for MALDI-MSI of dopamine and L-DOPA were prepared using a derivatization approach targeting primary amines and phenolic hydroxyl groups^27,48^. Slides were removed from −80 °C storage and warmed to room temperature in a desiccator over 30 min. The derivatization agent, reactive matrix FMP-10, was dissolved in 70% acetonitrile and 30% water (0.18 mg/ml) then sprayed across the tissue using the TM-sprayer in 30 passes at 80 °C (with pump flow rate 80 μL/min, nozzle velocity 1100 mm/min, 2.0 mm track spacing, and gas pressure 6 psi). After matrix application/derivatization, samples were placed in a MALDI slide adapter (Bruker Daltonics) and scanned using a Perfection V500 flatbed optical scanner (Epson, Japan).

### MALDI-MSI

MALDI-MSI analysis was performed using a solariX 7 T 2ω MALDI-FTICR MS system (Bruker Daltonics), equipped with a Smartbeam II 2 kHz laser, operated in positive ionization mode and calibrated with red phosphorus before analysis. An abundant phosphatidylcholine ion (PC (34:1)+K^+^, mass-to-charge (*m/z*) 798.540964 was used for online calibration during data acquisition. For MSI of neuropeptides, the lateral resolution was 100 µm and 100 laser shots were collected at each position. Data were acquired at *m/z* range of 450–5000 using a Q1 value of 650 *m/z*. The transfer optics time-of-flight and frequency were 1 ms and 4 MHz, respectively. For MSI of dopamine and L-DOPA, the lateral resolution was 150 µm and spectra were collected by firing 100 laser shots per pixel. The scanned *m/z* range was 150-1500 and the Q1 value was 378 *m/z*. The transfer optics time-of-flight and frequency were 0.7 ms and 4 MHz, respectively. Data were visualized using FlexImaging software (Bruker Daltonics, version 4.1). The spectra were subjected to root mean square (RMS) normalization.

### Statistical analysis

Prior to statistical analysis one animal in the non-LID group was excluded from the peptide analysis due to damage to its tissue section during sample preparation. SCiLS Lab software (Bruker Daltonics, version 2019b) was used to export average peak area values for neuropeptides, dopamine, and L-DOPA from specific brain regions of interest. These values were used for statistical analysis. Neuropeptide levels were imaged at the coronal brain level −6 mm from the anterior commissure (ac - 6 mm), focusing on the SN, Put, BCd, TCd, GPi, and GPe^51^. Dopamine and L-DOPA were imaged at −4 mm ac where their relative levels in the Put were determined for correlation analysis^51^.

SIMCA software (Sartorius Stedim Biotech, Umeå, Sweden, version 15.0) was used for multivariate data analysis. Prior to analysis, variables were auto-scaled (mean-centered and divided by the respective standard deviations). PCA was applied to obtain an overview of neuropeptide levels in each region of the basal ganglia of each treatment group. PLS-DA was performed to reveal specific differences between non-LID and LID groups. Neuropeptides with VIP values greater than 1 were considered to have the highest power for discriminating between the groups and subjected to further analysis. Hypothesis testing was performed using two non-parametric tests – the Mann–Whitney test when comparing two groups and the Kruskal–Wallis test for multi-group comparisons. FDR correction using the two-stage step-up method of Benjamini, Krieger, and Yekutieli^52^ with a *Q* value of 5% was performed in GraphPad Prism (Graphpad Software, La Jolla, California, USA, version 7.04). *Z*-scores were calculated to visualize differences in neuropeptide levels between groups using heatmaps. *Z*-scores were calculated using the equation *z* = (*x*−μ)/*σ*, where *x* is the mean peptide abundance of biological replicates in a group, μ is the mean peptide abundance in samples from all animals, and *σ* is the standard deviation of all samples. A heatmap showing the hierarchical clustering of neuropeptides in the GPi was generated using metaboanalyst^53^. Spearman’s correlation analysis was used to assess correlations between neuropeptide levels and dyskinesia scores, and Pearson’s correlation analysis was used to assess correlations between neuropeptide levels and those of dopamine and L-DOPA using GraphPad Prism. *P-*values from Spearman’s and Pearson’s correlation were adjusted using FDR. All analyses were performed with the significance threshold set at *P* = 0.05. Statistical test results are shown in Supplementary Tables 2–4.

### Reporting summary

Further information on research design is available in the Nature Research Reporting Summary linked to this article.

## Supplementary information

Reporting Summary

Supplementary Information

Supplementary Information

## Data Availability

The MALDI-MSI data supporting the findings of this study are available from the corresponding author upon request. Source data including the average peak area of the neuropeptides in all animals are available as Supplementary Data.
