# Supplementary material for: Basal ganglia neuropeptides show abnormal processing associated with L-DOPA-induced dyskinesia
Source: NPJ Parkinsons Dis. 2022 Apr 13;8:41. doi: 10.1038/s41531-022-00299-7 (PMC9007979; doi:10.1038/s41531-022-00299-7)
Supplement: Supplementary file 1 — Reporting Summary [file 41531_2022_299_MOESM1_ESM.pdf]

nature portfolio

Corresponding author(s): Per E. Andrén

Last updated by author(s): Feb 28, 2022

Reporting Summary

Nature Portfolio wishes to improve the reproducibility of the work that we publish. This form provides structure for consistency and transparency in reporting. For further information on Nature Portfolio policies, see our Editorial Policies and the Editorial Policy Checklist.

Statistics

For all statistical analyses, confirm that the following items are present in the figure legend, table legend, main text, or Methods section.

n/a	Confirmed	
☐	☒	The exact sample size (*n*) for each experimental group/condition, given as a discrete number and unit of measurement
☐	☒	A statement on whether measurements were taken from distinct samples or whether the same sample was measured repeatedly
☐	☒	The statistical test(s) used AND whether they are one- or two-sided<br>*Only common tests should be described solely by name; describe more complex techniques in the Methods section.*
☒	☐	A description of all covariates tested
☐	☒	A description of any assumptions or corrections, such as tests of normality and adjustment for multiple comparisons
☐	☒	A full description of the statistical parameters including central tendency (e.g. means) or other basic estimates (e.g. regression coefficient) AND variation (e.g. standard deviation) or associated estimates of uncertainty (e.g. confidence intervals)
☐	☒	For null hypothesis testing, the test statistic (e.g. *F*, *t*, *r*) with confidence intervals, effect sizes, degrees of freedom and *P* value noted<br>*Give P values as exact values whenever suitable.*
☒	☐	For Bayesian analysis, information on the choice of priors and Markov chain Monte Carlo settings
☒	☐	For hierarchical and complex designs, identification of the appropriate level for tests and full reporting of outcomes
☐	☒	Estimates of effect sizes (e.g. Cohen's *d*, Pearson's *r*), indicating how they were calculated

Our web collection on statistics for biologists contains articles on many of the points above.

Software and code

Policy information about availability of computer code

Data collection	MALDI MSI data: FTMS control (Bruker Daltonics, version 2.2.0)
Data analysis	FlexImaging (Bruker Daltonics, version 4.1), DataAnalysis (Bruker Daltonics, version 4.2), Scils lab (Bruker Daltonics, Bremen, v.2019c Pro), GraphPad Prism (v.7.04. GraphPad Software, La Jolla California USA). SIMCA, (v.15, Sartorius Stedim Biotech, Umeå, Sweden), RStudio (v.1.3.1056, RStudio Team, Boston, MA, USA).

For manuscripts utilizing custom algorithms or software that are central to the research but not yet described in published literature, software must be made available to editors and reviewers. We strongly encourage code deposition in a community repository (e.g. GitHub). See the Nature Portfolio guidelines for submitting code & software for further information.

Data

Policy information about availability of data

All manuscripts must include a data availability statement. This statement should provide the following information, where applicable:

- Accession codes, unique identifiers, or web links for publicly available datasets
- A description of any restrictions on data availability
- For clinical datasets or third party data, please ensure that the statement adheres to our policy

The MALDI-MSI data supporting the findings of this study are available from the corresponding author upon request. Source data including the average peak area of the neuropeptides in all animals are available online.

Field-specific reporting

Please select the one below that is the best fit for your research. If you are not sure, read the appropriate sections before making your selection.

☒ Life sciences ☐ Behavioural & social sciences ☐ Ecological, evolutionary & environmental sciences

For a reference copy of the document with all sections, see nature.com/documents/nr-reporting-summary-flat.pdf

Life sciences study design

All studies must disclose on these points even when the disclosure is negative.

Sample size	No sample size calculation were performed. Animal sample size was selected to minimize the number of sacrificed animals but to preserve the statistical power of quantitative work. Ethical concerns and sample availability were limiting factors for the number of samples that were chosen for primate brain samples.
Data exclusions	One sample was excluded in the non-LID group due to damages on its tissue section during sample preparation. One sample in the LID group was excluded prior to the statistical analysis since it did not cluster with other LID animals in a PCA.
Replication	Technical replicates of non-LID and LID animals were acquired on brain level -2 mm from anterior commisure
Randomization	Samples were analyzed in randomized order in and run in batches. Each batch contained 2 samples from each group.
Blinding	Blinding was not relevant to the study

Reporting for specific materials, systems and methods

We require information from authors about some types of materials, experimental systems and methods used in many studies. Here, indicate whether each material, system or method listed is relevant to your study. If you are not sure if a list item applies to your research, read the appropriate section before selecting a response.

Materials & experimental systems

n/a	Involved in the study	
☒	☐	Antibodies
☒	☐	Eukaryotic cell lines
☒	☐	Palaeontology and archaeology
☐	☒	Animals and other organisms
☒	☐	Human research participants
☒	☐	Clinical data
☒	☐	Dual use research of concern

Methods

n/a	Involved in the study	
☒	☐	ChIP-seq
☒	☐	Flow cytometry
☒	☐	MRI-based neuroimaging

Animals and other organisms

Policy information about studies involving animals; ARRIVE guidelines recommended for reporting animal research

Laboratory animals	Female rhesus monkeys (Macaca mulatta, Xierxin,mean age: 5 ± 1 years)
Wild animals	The study did not involve wild animals
Field-collected samples	The study did not involve field-collected samples
Ethics oversight	Primate tissue were obtained from a biobank (University of Bordeaux, France) where the previous experiments were carried out in an AAALAC-accredited facility in accordance with the European Communities Council Directive of November 24, 1986 (86/609/EEC) for care of laboratory animals.

Note that full information on the approval of the study protocol must also be provided in the manuscript.
