# Supplementary material for: Basal ganglia neuropeptides show abnormal processing associated with L-DOPA-induced dyskinesia
Source: NPJ Parkinsons Dis. 2022 Apr 13;8:41. doi: 10.1038/s41531-022-00299-7 (PMC9007979; doi:10.1038/s41531-022-00299-7)
Supplement: Supplementary file 2 — Supplementary Information [file 41531_2022_299_MOESM2_ESM.pdf]

Supplementary Information

Basal ganglia neuropeptides show abnormal processing associated with L-DOPA-induced dyskinesia

Heather Hulme[1,2a], Elva Fridjonsdottir[1a], Theodosia Vallianatou[1], Reza Shariatgorji[1,2], Anna Nilsson[1,2], Qin Li[3], Erwan Bezard[4,5,6], Per E. Andrén[1,2]*

1. Department of Pharmaceutical Biosciences, Medical Mass Spectrometry Imaging, Uppsala University, Uppsala, Sweden
2. Science for Life Laboratory, Spatial Mass Spectrometry, Uppsala University, Uppsala, Sweden,
3. Motac Neuroscience, Manchester, M15 6WE, United Kingdom,
4. Université de Bordeaux, Institut des Maladies Neurodégénératives, Bordeaux, France,
5. Centre National de la Recherche Scientifique, Institut des Maladies Neurodégénératives, Bordeaux, France,
6. Motac Neuroscience, Manchester, M15 6WE, United Kingdom.

[a] These authors contributed equally

*To whom correspondence should be addressed: per.andren@farmbio.uu.se

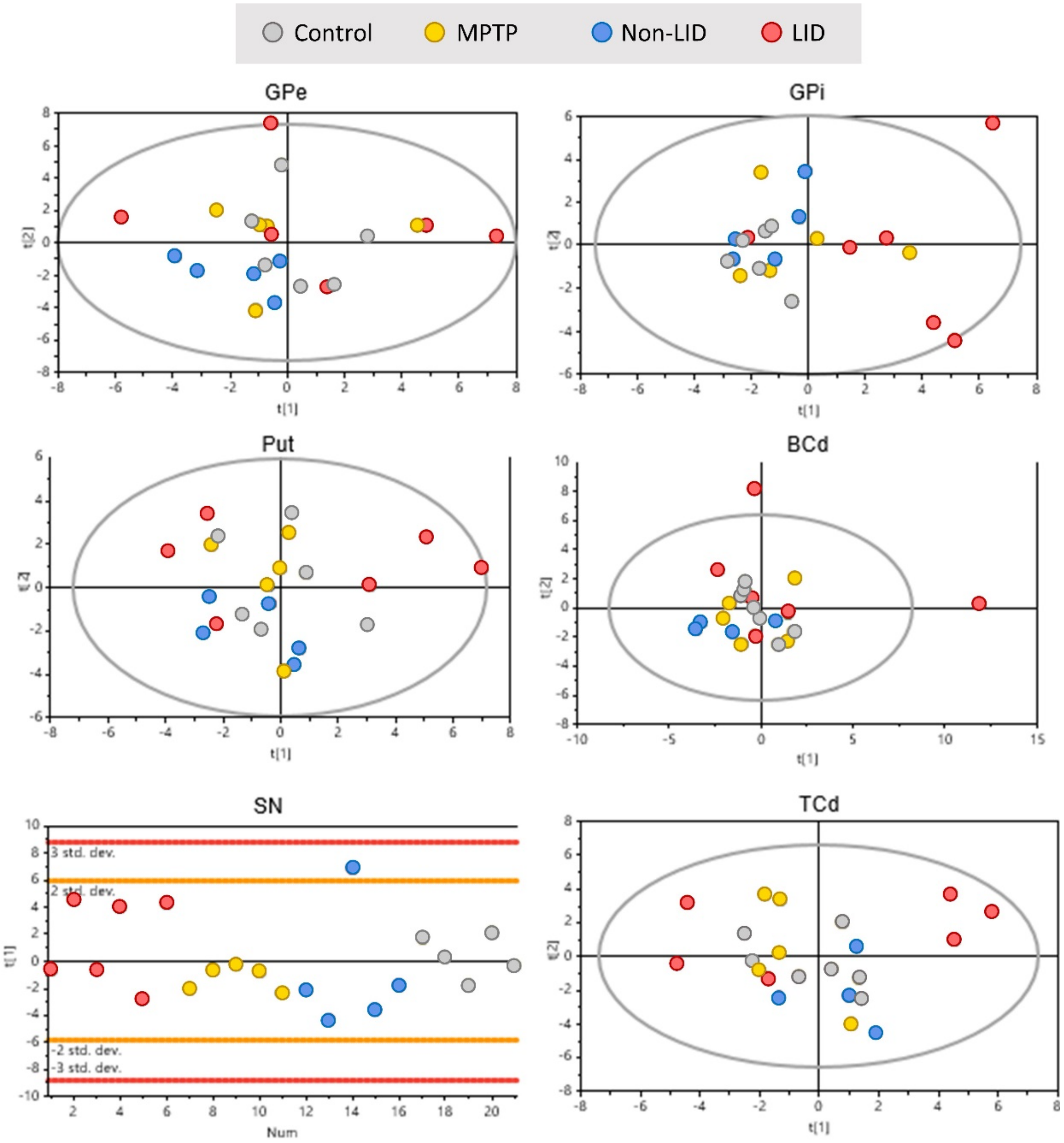

Supplementary Figure 1. Score plots from the PCA of the 23 detected neuropeptides in six basal ganglia regions.

The neuropeptides included in the PCA are listed in Supplementary Table 1. Annotations: BCd, body of the caudate; GPe, external globus pallidus; GPi, internal globus pallidus; Put, putamen; SN, substantia nigra; TCd, tail of the caudate.

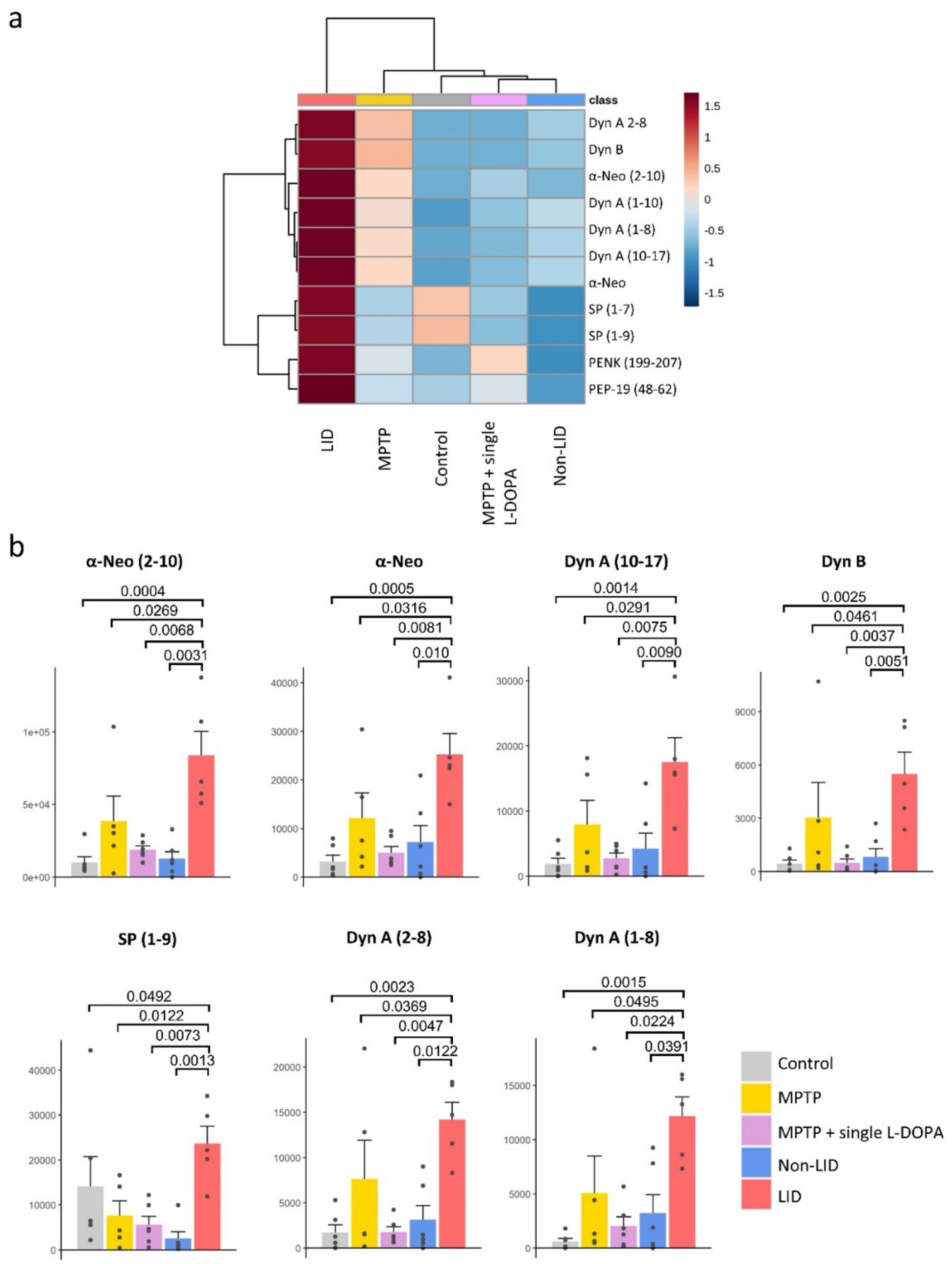

Supplementary Figure 2. Comparisons of neuropeptide levels in the GPi including results for MPTP-exposed animals that received a single L-DOPA dose.

(a) Relative abundances of the eleven neuropeptides most affected by LID in the GPi. Colors indicate auto-scaled averages for each group. The Euclidean distance measure and Ward clustering algorithm were used (ref number 48; Chong et. al. 2019). (b) Comparison of neuropeptide levels in the GPi based on the Kruskal-Wallis test followed by comparing the mean ranks of LID with the mean ranks of the remaining four groups. FDR correction was used for multiple comparisons; q-values are shown. Plots show the mean and standard deviation of the average peak areas of the peptides. n=6 for control and MPTP + single L-DOPA and n=5 for MPTP, non-LID and LID.

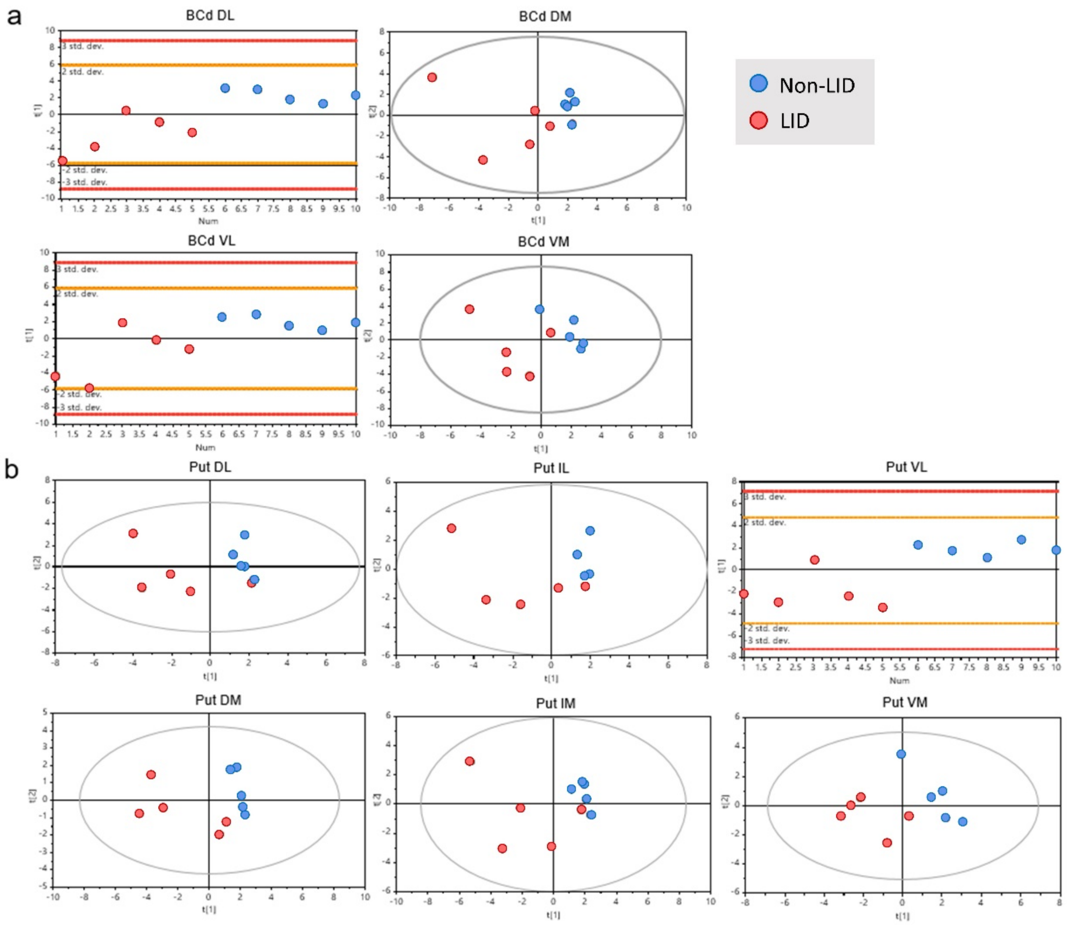

Supplementary Figure 3. **Separation of non-LID and LID animals by PLS-DA based on neuropeptides in sub-regions of the putamen and caudate.**

(a) PLS-DA score plots for sub-regions of BCd (b) PLS-DA score plots for sub-regions of Put. BCd, body of caudate; DL, Dorsolateral; DM, dorsomedial; IL, intermediolateral; IM, intermediomedial; Put, putamen; VL, ventrolateral; VM, ventromedial.

Supplementary Table 1. Neuropeptides detected using MALDI-MSI.

The precursor, amino acid sequence, theoretical and observed *m/z* values, and mass error in ppm are shown for each peptide.

Peptide precursor	Peptide name	Peptide sequence	Theoretical *m/z*	Observed *m/z*	Mass error [ppm]
Protachykinin-1	Substance P (1-11)	RPKPQQFFGLM-NH2	1347.735	1347.734	-0.519
	Substance P (1-9)	RPKPQQFFG-NH2	1104.594	1104.594	-0.091
	Substance P (1-7)	RPKPQQF	900.505	900.504	-1.110
	Neurokinin A	HKTDSFVGLM	1133.577	1133.575	-1.764
Proenkephalin	Leu-enk	YGGFL	556.277	556.278	1.852
	Met-enk	YGGFM	574.233	574.234	1.062
	Met-enk-Arg	YGGFMR	730.334	730.335	1.903
	Met-enk-Arg-Phe	YGGFMRF	877.403	877.402	-1.140
	Met-enk-Arg-Gly-Leu	YGGFMRGL	900.440	900.439	-1.111
	PENK (196-205)	SPQLEKEAKE	1145.532	1145.530	-1.833
	PENK (196-207)	SPQLEKEAKELQ	1386.675	1386.673	-1.442
	PENK (199-207)	LEKEAKELQ	1074.531	1074.531	<0.001
	PENK (217-227)	VGRPEWWMDYQ	1466.649	1466.650	0.682
	PENK (218-227)	GRPEWWMDYQ	1367.584	1367.580	-2.852
	PENK (219-227)	RPEWWMDYQ	1310.562	1310.559	-2.289
Prodynorphin	Dynorphin A (1-8)	YGGFLRRI	981.563	981.564	1.019
	Dynorphin A (2-8)	GGFLRRI	818.500	818.500	0.122
	Dynorphin A (10-17)	PKLKWDNQ	1028.552	1028.550	-1.944
	Dynorphin B (1-13)	YGGFLRRQFKVVT	1570.885	1570.882	-1.910
	a-neoendorphin (1-10)	YGGFLRKYPK	1228.684	1228.683	-0.814
	a-neoendorphin (2-10)	GGFLRKYPK	1065.620	1065.620	<0.001
	a-neoendorphin (3-10)	GFLRKYPK	1008.599	1008.598	-1.388
Calmodulin regulator protein PCP4	PEP-19 (48-62)	SQFRKFQKKKAGSQS	1754.966	1754.958	-4.558

Supplementary Table 2. Relative levels of neuropeptide abundances and statistical analysis.

VIP values from PLS-DA (only peptides with VIP>1 in at least one region are included), means ± standard deviation of ion intensity for each detected peptide in each group of animals, and q values (Mann-Whitney FDR-corrected p-values) from the comparison of non-LID and LID.

GPe	VIP	Control	MPTP	non-LID	LID	q value
Dyn A (2-8)	1.48441	6.174 ± 4.194	7.496 ± 6.142	1.631 ± 0.9738	9.366 ± 5.148	0.1232
Dyn A (1-8)	0.945747	4.462 ± 2.99	5.309 ± 5.069	2.24 ± 2.234	6.881 ± 6.248	
Dyn A (10-17)	1.2718	9.753 ± 4.071	12.57 ± 11.6	3.557 ± 1.526	15.35 ± 10.77	0.1232
Dyn B	1.10438	14.45 ± 5.32	15.6 ± 10.69	4.447 ± 2.093	18.08 ± 15.6	0.1232
α-neo (3-10)	0.759309	0.02874 ± 0.04549	0 ± 0	0 ± 0	0.254 ± 0.4763	
α-neo (2-10)	1.30043	28.56 ± 19.2	37.7 ± 23.5	11.93 ± 7.135	57.75 ± 40.02	0.0194
α-neo	0.886739	30.51 ± 12.64	33.79 ± 21.07	15.65 ± 7.903	36.21 ± 30.93	
Met-enk-R	0.378583	35.45 ± 9.347	35.73 ± 16.08	37.85 ± 8.111	30.77 ± 27.14	
PENK (199-207)	1.34174	95.2 ± 87.73	97.65 ± 40.59	66.73 ± 36.72	150.2 ± 59.48	0.0817
PENK (196-205)	1.09024	28.96 ± 20.63	24.35 ± 15.9	19.77 ± 12.75	42.07 ± 22.93	0.1232
PENK (219-227)	1.53076	180.9 ± 152.9	202.9 ± 151	95.68 ± 39.39	280.9 ± 109.4	0.0194
PENK (196-207)	0.680932	1144 ± 186.2	1101 ± 228.7	1001 ± 255.9	1265 ± 500.3	
SP (1-7)	1.35637	12.63 ± 7.797	9.83 ± 6.674	3.937 ± 3.957	13.75 ± 7.008	0.0582
SP (1-9)	1.58805	3.061 ± 5.113	1.319 ± 1.305	0.3497 ± 0.4003	2.97 ± 1.432	0.0194
PEP-19 (48-62)	1.09462	201 ± 171.4	323.4 ± 296.5	205.6 ± 80.71	660.1 ± 525.4	0.1633

GPi	VIP	Control	MPTP	non-LID	LID	q value
Dyn A (2-8)	1.4133	2.328 ± 2.832	10.34 ± 12.93	5.094 ± 5.286	19.2 ± 5.822	0.01
Dyn A (1-8)	1.28591	1.183 ± 1.319	9.847 ± 14.88	7.55 ± 8.429	23.68 ± 7.775	0.0219
Dyn A (10-17)	1.19287	4.032 ± 4.63	16.95 ± 17.68	10.91 ± 12.67	37.42 ± 17.98	0.0143
Dyn B	1.32819	2.181 ± 2.45	14.95 ± 21.8	4.792 ± 5.928	27.28 ± 13.59	0.01
α-neo (3-10)	0.949843	0 ± 0	0.05227 ± 0.1169	0.007498 ± 0.01677	4.286 ± 5.145	
α-neo (2-10)	1.40639	22.85 ± 22.34	88.61 ± 87.99	34.83 ± 25.32	192.1 ± 85.29	0.01
α-neo	1.23699	9.587 ± 9.794	36.77 ± 35.25	26.23 ± 25.48	76.62 ± 29.42	0.01
Met-enk-R		0.06509 ± 0.06787	0.2747 ± 0.3284	0.05849 ± 0.0616	0.08828 ± 0.1508	
PENK (199-207)	1.13292	0.4985 ± 0.615	0.8602 ± 0.6441	0.3529 ± 0.2411	1.962 ± 1.447	0.03
PENK (196-205)	0.614839	0.0412 ± 0.04994	0.04802 ± 0.08215	0.04452 ± 0.04253	0.08121 ± 0.06329	
PENK (219-227)	0.841124	0.5063 ± 0.3196	0.5827 ± 0.4136	0.5033 ± 0.3465	2.54 ± 2.872	
PENK (196-207)	0.416111	19.45 ± 4.591	26.88 ± 13.19	30.1 ± 28.4	40.98 ± 19.94	
SP (1-7)	1.30877	84.37 ± 58.65	54.43 ± 39.62	37.03 ± 18.07	133.7 ± 63.49	0.0143
SP (1-9)	1.49179	34.63 ± 39.75	18.61 ± 17.64	7.32 ± 9.525	57.99 ± 21.46	0.01
PEP-19 (48-62)	1.04905	298.9 ± 275.7	344.7 ± 352.3	177.9 ± 81.07	835 ± 675.9	0.03

SN	VIP	Control	MPTP	non-LID	LID	q value
Dyn A (2-8)	1.19607	240.9 ± 60.23	204.7 ± 36.41	159.8 ± 46.05	221.6 ± 33.87	0.0857
Dyn A (1-8)	1.05409	201.1 ± 75.75	194 ± 51.53	161.3 ± 59.25	278.6 ± 120.7	0.0857
Dyn A (10-17)	1.04675	352 ± 68.98	272.8 ± 58.04	230.1 ± 59.99	358.3 ± 134.7	0.1555
Dyn B	1.23417	502.3 ± 71.93	414.4 ± 76.98	300.7 ± 71.39	544.8 ± 201.7	0.0499
α-neo (3-10)	1.64788	30.01 ± 35.75	32.77 ± 19.94	4.677 ± 4.386	79.06 ± 38.67	0.0166
α-neo (2-10)	1.45402	757.7 ± 372.8	795.9 ± 378.8	448.7 ± 163.4	947.5 ± 288.9	0.0166
α-neo	0.921615	613.4 ± 231.6	501.9 ± 153	441.6 ± 131.7	689.8 ± 320.8	
Met-enk-R		0.6522 ± 0.5522	0.2023 ± 0.3718	1.93 ± 4.252	0.1762 ± 0.2676	
PENK (199-207)	0.509645	2.104 ± 2.454	0.2967 ± 0.4131	3.876 ± 8.644	6.284 ± 8.059	
PENK (196-205)	0.581849	0.6339 ± 0.9788	0.09001 ± 0.06096	1.437 ± 3.214	1.349 ± 1.685	
PENK (219-227)	0.558457	22.13 ± 24.19	13.9 ± 15.71	22.11 ± 44.39	47.32 ± 58.73	
PENK (196-207)	0.59972	212.3 ± 56.57	129.7 ± 40.64	227.9 ± 297	258.6 ± 107.8	
SP (1-7)	1.28444	140.8 ± 71.62	125.3 ± 36.19	91.07 ± 47.15	170.1 ± 44.05	0.0701
SP (1-9)	1.50753	49 ± 46.31	60.83 ± 52.22	30.98 ± 33.84	109.3 ± 27.34	0.0166
PEP-19 (48-62)	1.16446	276 ± 160.4	515.9 ± 258.6	330.7 ± 104.9	904 ± 565.9	0.1555

Put	VIP	Control	MPTP	non-LID	LID	q value
Dyn A (2-8)	0.676771	0.2979 ± 0.2835	0.3185 ± 0.2554	0.1831 ± 0.218	0.4393 ± 0.6185	
Dyn A (1-8)	0.610639	0.04751 ± 0.05283	0.03705 ± 0.02802	0.07376 ± 0.1084	0.1892 ± 0.2934	
Dyn A (10-17)	0.726991	0.5607 ± 0.426	0.4893 ± 0.3666	0.3692 ± 0.2864	0.8622 ± 1.1	
Dyn B	0.969498	1.767 ± 1.187	2.449 ± 1.685	0.754 ± 0.2619	2.445 ± 2.615	
α-neo (3-10)		0.001814 ± 0.002823	0 ± 0	0 ± 0	0.005616 ± 0.006439	
α-neo (2-10)	1.63785	7.66 ± 4.99	10.01 ± 7.249	4.26 ± 2.221	15.16 ± 6.015	0.0133
α-neo	0.720127	1.689 ± 1.574	1.311 ± 0.6724	1.24 ± 0.8621	2.394 ± 2.538	
Met-enk-R	1.23663	0.807 ± 0.6749	0.6461 ± 0.479	0.6611 ± 0.4685	0.2039 ± 0.2766	0.0195
PENK (199-207)	1.47019	3.617 ± 3.054	3.887 ± 1.798	2.075 ± 1.377	7.451 ± 4.016	0.0133
PENK (196-205)	0.905007	0.6711 ± 0.8971	0.2198 ± 0.1798	0.1728 ± 0.2215	0.4626 ± 0.4101	
PENK (219-227)	1.54865	15.64 ± 12.96	20.73 ± 20.26	5.483 ± 3.994	22.23 ± 10.68	0.0133
PENK (196-207)	0.60818	74.85 ± 21.23	78.01 ± 20.63	62.62 ± 24.14	69.77 ± 33.21	
SP (1-7)	1.38752	6.802 ± 5.249	5.149 ± 4.01	2.082 ± 1.237	5.988 ± 3.098	0.0133
SP (1-9)	1.63614	1.609 ± 3.034	0.4784 ± 0.2558	0.1096 ± 0.09016	0.8817 ± 0.4558	0.0133
PEP-19 (48-62)	1.1643	37.68 ± 38.85	62.92 ± 56.32	27 ± 26.19	179.3 ± 175.4	0.0286

BCd	VIP	Control	MPTP	non-LID	LID	q value
Dyn A (2-8)	0.786303	1.522 ± 1.061	1.373 ± 1.911	0.9077 ± 0.9022	1.826 ± 1.565	
Dyn A (1-8)	0.803822	0.7588 ± 0.5717	0.5382 ± 0.6295	0.4937 ± 0.542	3.064 ± 4.502	
Dyn A (10-17)	0.756642	2.162 ± 1.767	1.239 ± 0.8115	1.587 ± 1.951	3.729 ± 4.438	
Dyn B	0.804602	6.275 ± 3.833	5.201 ± 4.343	2.909 ± 1.924	9.927 ± 11.88	
α-neo (3-10)	1.10751	0.009779 ± 0.0158	0.07869 ± 0.176	0 ± 0	0.4301 ± 0.5958	0.0417
α-neo (2-10)	1.20699	22.65 ± 12.34	28.5 ± 25.23	11.38 ± 4.412	54.98 ± 38.26	0.0083
α-neo	0.790214	6.031 ± 4.611	3.641 ± 2.122	4.582 ± 5.009	11.65 ± 12.51	
Met-enk-R	1.09476	0.5733 ± 0.6914	0.5698 ± 0.7235	0.6772 ± 0.6197	0.2162 ± 0.2173	0.1625
PENK (199-207)	1.30031	7.661 ± 7.94	9.267 ± 6.305	2.927 ± 2.26	21.08 ± 12.5	0.0083
PENK (196-205)	1.8347	0.1803 ± 0.1254	0.09233 ± 0.08904	0.1386 ± 0.1232	0.443 ± 0.1206	0.0083
PENK (219-227)	1.26129	43.8 ± 33.22	59.3 ± 48.98	16.88 ± 11.9	82.16 ± 49.01	0.0083
PENK (196-207)	1.0294	147.4 ± 17.44	149.7 ± 46.61	122.9 ± 48.48	162 ± 72.42	0.1625
SP (1-7)	1.01866	12.59 ± 9.363	10.9 ± 8.178	4.651 ± 5.005	14.78 ± 10.19	0.0417
SP (1-9)	1.272	3.108 ± 4.943	1.16 ± 0.8545	0.1801 ± 0.1966	2.874 ± 1.96	0.0083
PEP-19 (48-62)	1.12661	77.47 ± 68.92	117.9 ± 86.54	38.57 ± 25.62	323.4 ± 348	0.1458

TCd	VIP	Control	MPTP	non-LID	LID	q value
Dyn A (2-8)	0.464737	2.635 ± 1.825	3.582 ± 1.983	2.417 ± 1.46	3.318 ± 2.964	
Dyn A (1-8)	0.689678	0.8063 ± 1.14	0.7609 ± 0.7023	0.8819 ± 0.8653	1.974 ± 2.476	
Dyn A (10-17)	0.394404	5.117 ± 3.308	3.448 ± 1.399	4.139 ± 1.806	5.873 ± 7.32	
Dyn B	0.516995	12.42 ± 6.737	12.52 ± 5.745	8.923 ± 4.419	12.97 ± 12.52	
α-neo (3-10)	1.48205	0.02656 ± 0.03046	0.01195 ± 0.02671	0 ± 0	0.2433 ± 0.2119	0.0055
α-neo (2-10)	1.48677	40.76 ± 14.65	59.09 ± 36.61	36.62 ± 18.54	70.28 ± 22.49	0.0111
α-neo	0.137161	13.28 ± 8.983	8.835 ± 5.043	14.31 ± 8.001	15.73 ± 16.14	
Met-enk-R	0.524164	4.406 ± 3.855	3.85 ± 2.467	4.478 ± 2.049	2.967 ± 4.429	
PENK (199-207)	1.88715	16.83 ± 16.55	14.14 ± 3.945	9.201 ± 5.269	23.28 ± 4.184	0.0055
PENK (196-205)	1.2063	1.411 ± 2.343	0.6614 ± 0.7424	0.5601 ± 0.7261	2.842 ± 2.663	0.0111
PENK (219-227)	1.54057	54.97 ± 49.51	66.16 ± 55.92	27.57 ± 20.33	70.98 ± 28.66	0.0111
PENK (196-207)	0.457203	245.7 ± 48.1	241.8 ± 71.75	209.8 ± 53.67	243.2 ± 112.5	
SP (1-7)	1.52305	6.87 ± 4.712	10.54 ± 5.02	6.506 ± 4.457	13.43 ± 3.598	0.0167
SP (1-9)	1.84327	2.423 ± 4.787	1.738 ± 1.815	0.4547 ± 0.3089	3.17 ± 1.375	0.0055
PEP-19 (48-62)	1.28518	55.82 ± 45.88	99.84 ± 71.31	37.17 ± 26.96	132.3 ± 101.4	0.0396

BCd DL	VIP	Control	MPTP	non-LID	LID	q value
Dyn A (2-8)	1.20466	0.1774 ± 0.2421	0 ± 0	0.01328 ± 0.0297	0.4657 ± 0.407	0.0175
Dyn A (1-8)	1.6667	0.01092 ± 0.02674	0.08577 ± 0.1918	0.04159 ± 0.09299	0.8007 ± 0.2534	0.005
Dyn A (10-17)	0.918718	0.3302 ± 0.3155	0.1646 ± 0.1845	0.3914 ± 0.8752	1.591 ± 1.376	
Dyn B	1.02637	1.241 ± 1.363	1.603 ± 2.119	0.4079 ± 0.3887	3.293 ± 3.338	0.005
α-neo (3-10)		0.01552 ± 0.03803	0 ± 0	0 ± 0	0.8162 ± 1.776	
α-neo (2-10)	1.11019	12.74 ± 16.25	13.81 ± 18.61	4.453 ± 1.553	72.17 ± 69.99	0.005
α-neo	1.49279	1.243 ± 1.167	0.5235 ± 0.5541	0.9488 ± 1.562	5.749 ± 2.178	0.0083
Met-enk-R	0.71253	0.06247 ± 0.1269	0.2938 ± 0.657	0.2108 ± 0.342	0.02636 ± 0.04005	
PENK (199-207)	1.20237	3.236 ± 4.55	6.599 ± 4.172	2.321 ± 2.065	29.34 ± 24.37	0.005
PENK (196-205)	0.73243	0.0758 ± 0.12	0.06709 ± 0.09415	0.1077 ± 0.1328	0.3539 ± 0.4247	
PENK (219-227)	1.28538	22.1 ± 19.64	47.2 ± 41.12	8.466 ± 5.185	94.8 ± 68.79	0.005
SP (1-7)	1.12766	12.15 ± 12.49	2.541 ± 2.416	1.679 ± 1.892	9.937 ± 8.108	0.0175
SP (1-9)	1.04499	2.594 ± 4.934	0.3029 ± 0.4943	0.08393 ± 0.1327	1.167 ± 1.221	0.0143
PEP-19 (48-62)	1.01561	77.23 ± 79.76	115.8 ± 109.8	35.89 ± 25.05	456.9 ± 497.3	0.0175

BCd DM	VIP	Control	MPTP	non-LID	LID	q value
Dyn A (2-8)	0.852038	1.743 ± 1.575	0.4271 ± 0.8319	0.5258 ± 0.7211	2.013 ± 2.134	
Dyn A (1-8)	0.837283	0.6283 ± 0.5997	0.5969 ± 0.6172	0.4094 ± 0.7054	4.97 ± 9.194	
Dyn A (10-17)	0.731637	2.36 ± 1.872	0.9199 ± 0.8126	1.461 ± 2.696	5.103 ± 7.583	
Dyn B	0.866942	7.189 ± 2.823	5.765 ± 3.629	2.622 ± 1.948	13.9 ± 19.61	
α-neo (3-10)	1.00049	0 ± 0	0 ± 0	0 ± 0	0.203 ± 0.2461	0.05
α-neo (2-10)	1.27307	36.27 ± 20.53	35.15 ± 24.29	16.31 ± 10.97	57.15 ± 31.71	0.05
α-neo	0.81235	6.491 ± 3.165	3.842 ± 2.099	4.074 ± 6.148	16.09 ± 22.58	
Met-enk-R	1.29527	0.6768 ± 0.9979	0.4728 ± 0.9374	0.4858 ± 0.4932	0.02538 ± 0.0383	0.05
PENK (199-207)	1.57862	11.47 ± 11.28	11.47 ± 8.164	2.245 ± 1.402	19.07 ± 9.301	0.0249
PENK (196-205)	1.42025	0.211 ± 0.2008	0.09643 ± 0.1432	0.1025 ± 0.1454	0.5381 ± 0.3526	0.05
PENK (219-227)	1.28228	72.78 ± 54.83	77.47 ± 57.03	25.02 ± 21.51	94.5 ± 54.23	0.05
SP (1-7)	1.03926	17.25 ± 12.63	16.12 ± 14.62	6.267 ± 6.876	20.92 ± 16.39	0.05
SP (1-9)	1.34765	4.795 ± 7.065	1.476 ± 0.9489	0.04989 ± 0.1116	4.386 ± 3.25	0.0249
PEP-19 (48-62)	0.999151	88.7 ± 67.83	145.8 ± 101.9	44.93 ± 34.89	249 ± 258.6	

BCd VL	VIP	Control	MPTP	non-LID	LID	q value
Dyn A (2-8)		0.04359 ± 0.1068	0.006135 ± 0.01372	0.005884 ± 0.01316	0.118 ± 0.2027	
Dyn A (1-8)	0.957	0 ± 0	0 ± 0	0 ± 0	0.3969 ± 0.6026	
Dyn A (10-17)	0.905839	0.1938 ± 0.2521	0.06929 ± 0.09575	0.1595 ± 0.3566	0.8908 ± 1.135	
Dyn B	0.966325	1.755 ± 1.785	1.694 ± 1.669	0.158 ± 0.2467	2.013 ± 2.772	
α-neo (3-10)		0 ± 0	0 ± 0	0 ± 0	0.06606 ± 0.1264	
α-neo (2-10)	1.27428	6.249 ± 8.623	9.26 ± 11.17	1.601 ± 1.079	35.77 ± 34.63	0.0028
α-neo	1.17618	0.696 ± 0.8403	0.6584 ± 0.3915	0.5368 ± 0.7586	4.091 ± 4.004	0.0055
Met-enk-R		0.01621 ± 0.02834	0.09862 ± 0.2205	0.03439 ± 0.05477	0.01899 ± 0.04246	
PENK (199-207)	1.30986	2.218 ± 2.395	4.939 ± 4.044	2.242 ± 1.737	19.67 ± 16.8	0.0028
PENK (196-205)	0.785829	0.05945 ± 0.1456	0.0785 ± 0.0928	0.03712 ± 0.08301	0.1034 ± 0.09711	
PENK (219-227)	1.37548	14.69 ± 13.68	27.59 ± 26.46	5.912 ± 4.319	62.43 ± 50.17	0.0042
SP (1-7)	1.24546	8.525 ± 8.306	5.241 ± 5.598	1.152 ± 1.363	10.05 ± 9.251	0.0055
SP (1-9)	1.26768	1.903 ± 3.356	0.5618 ± 0.3892	0.03128 ± 0.04327	1.885 ± 1.895	0.0028
PEP-19 (48-62)	1.1068	81.35 ± 74.57	110.5 ± 76.47	44.89 ± 29.37	410.1 ± 456.1	0.0333

BCd VM	VIP	Control	MPTP	non-LID	LID	q value
Dyn A (2-8)	0.774762	4.777 ± 3.863	3.825 ± 3.44	3.245 ± 3.865	5.138 ± 4.134	
Dyn A (1-8)	0.884854	2.726 ± 2.371	1.609 ± 1.875	1.619 ± 1.432	6.614 ± 9.007	
Dyn A (10-17)	0.697621	6.53 ± 6.045	3.93 ± 2.549	4.568 ± 5.091	8.198 ± 9.111	
Dyn B	0.892053	17.03 ± 13.36	11.79 ± 9.085	9.175 ± 7.825	22.59 ± 23.96	
α-neo (3-10)	1.35805	0.02832 ± 0.06937	0.2487 ± 0.5562	0 ± 0	0.7431 ± 0.7324	0.09
α-neo (2-10)	1.38822	41.41 ± 13.53	54.25 ± 36.98	26.21 ± 16.29	61.71 ± 28.04	0.0749
α-neo	0.64664	17.75 ± 16.35	10.24 ± 5.225	13.56 ± 12.77	23.09 ± 24.27	
Met-enk-R	1.13566	1.766 ± 2.057	1.334 ± 1.212	2.171 ± 2.359	0.8374 ± 0.8255	0.4001
PENK (199-207)	1.49545	16.33 ± 17.21	16.02 ± 10.94	5.493 ± 6.06	19.33 ± 9.219	0.0749
PENK (196-205)	1.01446	0.4526 ± 0.3603	0.1468 ± 0.1347	0.3102 ± 0.3453	0.8925 ± 0.7715	0.1375
PENK (219-227)	1.62669	78.71 ± 60.48	89.64 ± 70.36	32.23 ± 22.55	87.41 ± 29.83	0.0749
SP (1-7)	1.00226	15.98 ± 7.987	23.75 ± 16.27	11.09 ± 11.55	20.91 ± 11.66	0.2036
SP (1-9)	1.45141	4.385 ± 6.335	2.31 ± 1.734	0.644 ± 0.6614	4.305 ± 3.244	0.0749
PEP-19 (48-62)	1.23493	72.85 ± 62.05	109.6 ± 71.43	29.28 ± 21.25	195.1 ± 195.9	0.2625

Put DL	VIP	Control	MPTP	non-LID	LID	q value
α-neo (2-10)	1.51925	0.3498 ± 0.6661	1.283 ± 2.159	0.1613 ± 0.3105	9.408 ± 6.397	0.1002
α-neo	0.849087	0.06246 ± 0.04757	0.08993 ± 0.06375	0.07242 ± 0.08668	0.8269 ± 1.332	
Dyn B	0.966099	0.3975 ± 0.2923	0.8123 ± 0.717	0.201 ± 0.1433	0.6439 ± 0.6426	
Met-enk-RF	0.738254	1.496 ± 0.8801	1.483 ± 1.76	4.63 ± 3.483	5.094 ± 5.901	
PENK (199-207)	1.03126	0.09361 ± 0.09328	0.332 ± 0.2527	1.03 ± 1.055	3.959 ± 3.742	0.2333
PENK (196-205)	0.817716	0.03592 ± 0.04301	0.009217 ± 0.02061	0.01725 ± 0.01836	0.06991 ± 0.09599	
PENK (219-227)	1.46004	1.91 ± 2.101	4.763 ± 5.413	1.643 ± 1.264	11.6 ± 7.751	0.12
PENK (196-207)	0.89428	21.68 ± 11.58	25.15 ± 10.76	31.79 ± 21.81	36.25 ± 24.85	
SP (1-7)	1.2427	4.348 ± 4.349	1.014 ± 1.306	0.1591 ± 0.1918	2.412 ± 2.282	0.12
SP (1-9)	1.15028	0.7641 ± 1.543	0.09779 ± 0.07276	0.05033 ± 0.04419	0.246 ± 0.2185	0.12
PEP-19 (48-62)	1.11922	32.31 ± 33.59	61.61 ± 63.67	25.54 ± 26.45	206.2 ± 218.4	0.12

Put DM	VIP	Control	MPTP	non-LID	LID	q value
α-neo (2-10)	1.11782	0.9078 ± 1.44	2.061 ± 3.605	0.1726 ± 0.2414	7.682 ± 6.775	0.0166
α-neo	1.10959	0.06179 ± 0.06652	0.06488 ± 0.06523	0.05715 ± 0.05388	0.6212 ± 0.5187	0.0389
Dyn B	1.07005	0.3881 ± 0.4515	0.8372 ± 0.7704	0.1659 ± 0.1455	0.9243 ± 0.7459	0.0223
Met-enk-RF	0.72248	5.071 ± 3.225	5.562 ± 2.748	8.446 ± 5.874	9.072 ± 8.637	
PENK (199-207)	0.866021	0.7382 ± 0.8292	2.453 ± 3.411	1.173 ± 1.353	6.106 ± 7.262	
PENK (196-205)	0.971013	0.05649 ± 0.06754	0.02455 ± 0.02353	0.02295 ± 0.03553	0.1325 ± 0.134	
PENK (219-227)	1.10318	4.27 ± 4.07	17.3 ± 22.37	2.156 ± 1.788	19.77 ± 17.68	0.0389
PENK (196-207)	1.10392	45.52 ± 19.66	51.32 ± 29.39	40.83 ± 26.58	60.58 ± 43.55	0.3286
SP (1-7)	0.810743	4.538 ± 4.265	3.08 ± 3.64	0.3962 ± 0.6696	3.007 ± 3.64	
SP (1-9)	1.68966	0.5061 ± 1.081	0.2306 ± 0.256	0.02358 ± 0.02586	0.3141 ± 0.1264	0.0166
PEP-19 (48-62)	1.08739	46.44 ± 42.82	83.43 ± 91.83	31.55 ± 32.79	255.2 ± 245.5	0.0333

Put IL	VIP	Control	MPTP	non-LID	LID	q value
α-neo (2-10)	1.16936	0.1262 ± 0.2263	1.174 ± 1.722	0.03744 ± 0.05106	3.085 ± 2.738	0.0223
α-neo	0.966295	0.04246 ± 0.03643	0.06285 ± 0.02406	0.04324 ± 0.03982	0.3735 ± 0.3336	
Dyn B	0.818136	0.7312 ± 0.5351	1.572 ± 1.098	0.3091 ± 0.2999	1.593 ± 2.086	
Met-enk-RF	1.02444	1.362 ± 0.7465	1.586 ± 1.723	4.392 ± 2.849	6.751 ± 6.675	0.5999
PENK (199-207)	1.07913	0.0785 ± 0.07697	0.4422 ± 0.349	0.6304 ± 0.513	3.134 ± 2.834	0.26
PENK (196-205)	0.815238	0.03663 ± 0.07077	0.03286 ± 0.03855	0.01086 ± 0.01793	0.06335 ± 0.09735	
PENK (219-227)	1.15408	1.589 ± 1.559	4.752 ± 5.522	1.08 ± 0.8347	7.836 ± 6.662	0.2333
PENK (196-207)	1.02344	20.25 ± 11.39	26.87 ± 10.49	26.11 ± 17.01	25.71 ± 17.6	0.5999
SP (1-7)	1.30039	5.123 ± 5.469	1.221 ± 1.525	0.1557 ± 0.2288	2.681 ± 1.978	0.0223
SP (1-9)	1.31744	1.571 ± 3.089	0.1265 ± 0.1174	0.03599 ± 0.04938	0.6527 ± 0.6035	0.0223
PEP-19 (48-62)	0.972233	28.07 ± 31.47	45.15 ± 40.63	23.71 ± 23.5	130.4 ± 127.4	

Put IM	VIP	Control	MPTP	non-LID	LID	q value
α-neo (2-10)	1.31881	0.6235 ± 0.8994	1.64 ± 1.926	0.1884 ± 0.3186	4.384 ± 2.834	0.0747
α-neo	1.07734	0.1058 ± 0.1352	0.2824 ± 0.1324	0.06748 ± 0.1	0.7017 ± 0.792	0.075
Dyn B	0.771117	0.7714 ± 0.396	1.501 ± 1.008	0.186 ± 0.2385	0.6761 ± 0.9763	
Met-enk-RF	0.680945	4.458 ± 2.388	5.058 ± 3.908	7.951 ± 5.046	9.189 ± 8.067	
PENK (199-207)	1.12706	0.7241 ± 0.6996	1.611 ± 1.268	0.787 ± 0.6573	4.723 ± 3.578	0.0749
PENK (196-205)	1.05147	0.03144 ± 0.04895	0.03852 ± 0.05417	0.004188 ± 0.009365	0.09965 ± 0.1317	0.075
PENK (219-227)	1.28986	4.317 ± 3.55	11.9 ± 16.41	1.294 ± 1.018	10.91 ± 6.713	0.0751
PENK (196-207)	1.17532	40.12 ± 11.76	51.37 ± 17.91	37.33 ± 16.89	45.3 ± 29.71	0.8834
SP (1-7)	1.05301	6.005 ± 6.026	3.882 ± 4.839	0.6004 ± 1.006	3.484 ± 2.848	0.0749
SP (1-9)	1.13495	1.276 ± 2.326	0.323 ± 0.2576	0.01983 ± 0.02898	0.5114 ± 0.4511	0.0749
PEP-19 (48-62)	1.22861	39.71 ± 48.21	50.94 ± 41.8	24.56 ± 26.09	176.5 ± 164	0.1125

Put VL	VIP	Control	MPTP	non-LID	LID	q value
α-neo (2-10)	1.64598	18.58 ± 13.05	20.48 ± 13.63	8.548 ± 3.123	34.29 ± 12.2	0.0111
α-neo	0.728084	2.033 ± 1.657	1.183 ± 0.6592	1.691 ± 1.546	5.398 ± 7.054	
Dyn B	0.980143	2.927 ± 2.139	2.962 ± 1.68	1.02 ± 0.5883	4.301 ± 4.379	
Met-enk-RF	0.388307	22.16 ± 8.046	15.06 ± 7.486	22.56 ± 10.36	28.97 ± 22.44	
PENK (199-207)	1.68714	6.27 ± 7.33	6.361 ± 3.415	2.672 ± 1.75	10.62 ± 3.071	0.0111
PENK (196-205)	0.998445	0.4888 ± 0.7571	0.5512 ± 0.676	0.0856 ± 0.04882	0.3259 ± 0.3117	0.0619
PENK (219-227)	1.36221	36.71 ± 31.48	39.72 ± 34.71	11.49 ± 8.112	36.22 ± 18	0.0467
PENK (196-207)	0.406317	134.2 ± 27.35	134.1 ± 18.55	95.52 ± 27.78	107.9 ± 36.06	
SP (1-7)	1.48925	7.566 ± 6.233	5.154 ± 4.205	2.679 ± 1.562	10.81 ± 5.104	0.0111
SP (1-9)	1.13204	2.255 ± 4.707	0.3393 ± 0.2043	0.09169 ± 0.09087	1.011 ± 1.005	0.0333
PEP-19 (48-62)	1.05722	49.88 ± 43.72	81.31 ± 51.08	35.19 ± 31.49	162.8 ± 151.5	0.0619

Put VM	VIP	Control	MPTP	non-LID	LID	q value
α-neo (2-10)	1.34853	36.24 ± 19.39	45.41 ± 31.59	24.64 ± 16.26	49.12 ±13.05	0.0584
α-neo	0.723361	10.21 ± 10.31	7.505 ± 4.68	7.139 ± 5.481	8.68 ± 9.018	
Dyn B	0.750216	7.25 ± 5.279	9.25 ± 6.388	3.133 ± 1.043	7.93 ± 9.391	
Met-enk-RF	0.957583	44.35 ± 14.42	34.26 ± 11.83	43.44 ± 26.06	38.12 ± 14.25	
PENK (199-207)	1.6789	19.48 ± 15.38	15.98 ± 6.559	8.574 ± 5.281	23.65 ± 7.903	0.0417
PENK (196-205)	0.699482	4.673 ± 6.61	0.8551 ± 0.6951	1.125 ± 1.61	2.866 ± 3.283	
PENK (219-227)	1.5487	66.34 ± 54.75	67.88 ± 60.96	23.82 ± 18.04	69.84 ± 22.84	0.0555
PENK (196-207)	0.522298	264.5 ± 82.03	243.6 ± 59.8	202.4 ± 60.64	208.3 ± 61.95	
SP (1-7)	1.16539	17.33 ± 8.296	23.12 ± 14.81	12.46 ± 7.049	20.8 ± 5.969	0.1944
SP (1-9)	1.33529	4.079 ± 7.06	2.158 ± 1.169	0.4706 ± 0.3911	3.171 ± 2.308	0.0415
PEP-19 (48-62)	1.15859	32.5 ± 36.11	47.98 ± 31.56	17.41 ± 18.36	120.9 ± 112.4	0.0584

Supplementary Table 3. Results of Spearman's correlation analysis of relationships between neuropeptide abundances and LID scores.

Spearman correlation coefficient (r) and FDR adjusted q-values for each correlation is shown.

	GPe		GPi		BCd		Put		SN		TCd	
	r	*q-value*	*r*	*q-value*	*r*	*q-value*	*r*	*q-value*	*r*	*q-value*	*r*	*q-value*
Leu-enk	-0.23	0.7001										
Met-enk	-0.21	0.7099	-0.13	0.8362	0.25	0.5282	-0.53	0.3394	0.03	0.9853	-0.11	0.9309
Met-enk-R	-0.07	0.9152	-0.01	0.9921	-0.34	0.4561	-0.57	0.2844	-0.15	0.7885	-0.26	0.9309
Met-enk-RF	-0.06	0.9152	-0.03	0.9921	0.25	0.5282	0.11	0.9778	0.22	0.6626	0.09	0.9309
Met-enk-RGL	0.06	0.9152	-0.18	0.7915	0.25	0.5282	0.01	0.9937	0.1	0.8660	-0.15	0.9309
PENK (199-207)	0.57	0.2592	0.38	0.4647	0.86	0.0220	0.68	0.1368	0.59	0.2769	0.86	0.0579
PENK (196-205)	0.46	0.2974	0.24	0.7269	0.76	0.0525	0.41	0.5291	0.27	0.5811	0.68	0.1072
PENK (219-227)	0.86	0.0307	0.39	0.4647	0.89	0.0176	0.78	0.0699	0.53	0.3202	0.82	0.0579
PENK (218-227)	0.55	0.2592	0.16	0.7915	0.16	0.6926	0.05	0.9778	0.42	0.3828	0.14	0.9309
PENK (196-207)	0.47	0.2974	0.36	0.4889	0.55	0.2339	0.22	0.9117	0.42	0.3828	0.26	0.9309
PENK (217-227)	0.04	0.9214	0.17	0.7915	0.25	0.5282	0.11	0.9778	0.22	0.6626	-0.05	0.9516
Dyn A (2-8)	0.66	0.2079	0.8	0.0418	0.4	0.4153	0.09	0.9778	0.51	0.3202	0.09	0.9309
Dyn A (1-8)	0.41	0.3688	0.47	0.3492	0.55	0.2339	0.07	0.9778	0.48	0.3509	0.15	0.9309
Dyn A (10-17)	0.56	0.2592	0.72	0.0773	0.37	0.4296	0.01	0.9937	0.38	0.4427	-0.03	0.9516
Dyn B	0.63	0.2281	0.84	0.0308	0.47	0.3366	0.16	0.9696	0.65	0.2094	0.03	0.9516
a-neo (3-10)	0.61	0.2614	0.85	0.0308	0.82	0.0418	0.57	0.3464	0.79	0.0814	0.88	0.0579
a-neo (2-10)	0.92	0.0184	0.89	0.0176	0.92	0.0176	0.87	0.0528	0.89	0.0352	0.78	0.0699
a-neo	0.3	0.5625	0.7	0.0775	0.39	0.4153	0.2	0.9117	0.45	0.3828	-0.11	0.9309
SP (1-7)	0.82	0.0454	0.78	0.0466	0.76	0.0525	0.82	0.0699	0.7	0.1744	0.7	0.1072
SP (1-9)	0.89	0.0184	0.89	0.0176	0.86	0.0220	0.79	0.0699	0.79	0.0814	0.76	0.0735
SP (1-11)	0.49	0.2974	0.54	0.2479	0.4	0.4153	0.35	0.6396	0.3	0.5739	0.26	0.9309
NK A	0.11	0.9152	0.03	0.9921	0.03	0.9516	-0.2	0.9117	-0.01	0.9937	-0.14	0.9309
PEP-19 (48-62)	0.54	0.2592	0.7	0.0775	0.57	0.2339	0.68	0.1368	0.57	0.2769	0.63	0.1636

Supplementary Table 4. Results of Pearsons's correlation analysis of relationships between neuropeptide abundances in GPi, GPe and SN with putaminal dopamine and L-DOPA levels.

Pearsons's correlation coefficient (r) and FDR adjusted q-values for each correlation is shown.

Correlation w. putaminal dopamine	GPe		GPi		SN	
	r	*q-value*	*r*	*q-value*	*r*	*q-value*
Leu-enk	-0.245	0.9964				
Met-enk	-0.00154	0.9964	-0.292	0.8925	-0.211	0.7014
Met-enk-R	0.0105	0.9964	0.606	0.8925	-0.24	0.7014
Met-enk-RF	0.0957	0.9964	-0.105	0.8925	0.108	0.8764
Met-enk-RGL	0.166	0.9964	0.382	0.8925	-0.0164	0.9761
PENK (199-207)	0.0388	0.9964	0.132	0.8925	-0.243	0.7014
PENK (196-205)	-0.0551	0.9964	0.247	0.8925	-0.269	0.7014
PENK (219-227)	0.0546	0.9964	-0.175	0.8925	-0.242	0.7014
PENK (218-227)	0.0489	0.9964	-0.016	0.9628	-0.156	0.7998529
PENK (196-207)	0.0402	0.9964	-0.13	0.8925	-0.0822	0.8953737
PENK (217-227)	0.363	0.74855	-0.0458	0.938385	0.0103	0.9761
Dyn A (2-8)	0.569	0.5028571	0.485	0.8925	0.31	0.7014
Dyn A (1-8)	0.475	0.5028571	0.302	0.8925	0.654	0.30345
Dyn A (10-17)	0.465	0.5028571	0.343	0.8925	0.537	0.50022
Dyn B	0.49	0.5028571	0.452	0.8925	0.667	0.30345
a-neo (3-10)	-0.146	0.9964	-0.2	0.8925	0.498	0.50022
a-neo (2-10)	0.138	0.9964	0.121	0.8925	0.276	0.7014
a-neo	0.48	0.5028571	0.343	0.8925	0.522	0.50022
SP (1-7)	0.54	0.5028571	0.069	0.9286421	0.213	0.7014
SP (1-9)	0.0148	0.9964	0.102	0.8925	0.334	0.7014
SP (1-11)	0.455	0.5028571	0.36	0.8925	0.394	0.7014
NK A	0.321	0.8206	0.137	0.8925	0.346	0.7014

Correlation w. putaminal L-DOPA	GPe		GPi		SN	
	r	*q-value*	*r*	*q-value*	*r*	*q-value*
Leu-enk	0.1152596	0.7708056				
Met-enk	0.358267	0.3623309	-0.0754	0.868875	0.0789	0.8408
Met-enk-R	0.1365856	0.7577075	0.0746	0.868875	-0.0982	0.8408
Met-enk-RF	0.5200978	0.2469011	0.684	0.0707	0.332	0.7443333
Met-enk-RGL	0.4546751	0.320022	0.549	0.16863	0.294	0.79863
PENK (199-207)	0.4314342	0.3395242	0.135	0.8085	-0.118	0.8408
PENK (196-205)	0.6170877	0.1197268	0.135	0.8085	0.0967	0.8408
PENK (219-227)	0.3647531	0.3623309	0.0266	0.938	-0.166	0.8408
PENK (218-227)	0.4070834	0.3621792	0.635	0.0945	0.0687	0.8408
PENK (196-207)	0.3577976	0.3623309	0.469	0.25445	0.17	0.8408
PENK (217-227)	0.6161323	0.1197268	0.616	0.1017333	0.126	0.8408
Dyn A (2-8)	0.3932606	0.3623309	0.641	0.0945	0.428	0.5658
Dyn A (1-8)	0.8456401	0.00572	0.507	0.2124818	0.638	0.14574
Dyn A (10-17)	0.9189051	0.001408	0.867	0.012075	0.824	0.0189
Dyn B	0.8740438	0.00319	0.824	0.012075	0.759	0.0469
a-neo (3-10)	-0.09190969	0.788111	-0.142	0.8085	0.191	0.8408
a-neo (2-10)	0.2256341	0.6168666	0.184	0.8085	0.132	0.8408
a-neo	0.779904	0.0170097	0.816	0.012075	0.834	0.0189
SP (1-7)	0.4892275	0.2787422	0.268	0.63915	0.226	0.8408
SP (1-9)	0.173316	0.7066747	0.324	0.5355	0.341	0.7443333
SP (1-11)	0.8232016	0.0081444	0.774	0.02184	0.568	0.238
NK A	0.889803	0.002684	0.813	0.012075	0.68	0.111825

Supplementary Note.

Sub-regional analysis of neuropeptides.

The neuropeptides' distributions varied across the analyzed regions. Previous studies on the functional anatomy of the striatum have defined sub-regions of the Put and BCd according to the areas of the cortex they are connected to and their roles. Therefore, we tested the hypothesis that the observed treatment-related elevations in neuropeptide levels may have been particularly strong in specific sub-regions of the striatal brain areas, Put, and BCd (Fig. 4a). To this end, we subjected the data on peptide levels in all sub-regions to PLS-DA (Supplementary Fig. 3) and used the Mann-Whitney test to evaluate the significance of between-group differences in the levels of peptides for which VIP>1. In the BCd, significant between-group differences were primarily found in the dorsolateral (DL) and ventrolateral (VL) sub-regions (Fig. 3b). Levels of seven neuropeptides were higher in these regions in the LID group than in the non-LID group, on average, across the whole BCd (Fig. 2b). However, levels of 10 neuropeptides were significantly higher in the DL BCd compared to only seven in the VL BCd. In addition, only two peptides exhibited significant between-group differences in the DM BCd and none in the VM BCd, showing that the treatments affected neuropeptides more strongly in the lateral part of the BCd than in its medial part.

We also detected subregion-specific differences in the levels of several neuropeptides in the putamen, mostly in the dorsomedial (DM) and ventromedial (VM) sub-regions (Fig. 4c). Dyn B, α-neo (2-10), α-neo, PENK (219-227), SP (1-9), and PEP-19 (48-62) were more abundant in LID than in non-LID animals in the DM Put. Additionally, in the VM Put, α-neo (2-10), PENK (199-207), PENK (219-227), SP (1-7), and SP (1-9) were more abundant in LID than in non-LID animals. Finally, levels of α-neo (2-10), SP (1-7), and SP (1-9) were significantly higher in LID animals in the intermediolateral (IL) part of the Put.
